# Distinctive reactivity of *N*-benzylidene-[1,1'-biphenyl]-2-amines under photoredox conditions

**DOI:** 10.3762/bjoc.16.114

**Published:** 2020-06-18

**Authors:** Shrikant D Tambe, Kwan Hong Min, Naeem Iqbal, Eun Jin Cho

**Affiliations:** 1 Department of Chemistry, Chung-Ang University, 84 Heukseok-ro, Dongjak-gu, Seoul 06974, Republic of Korea

**Keywords:** 1,2-diamine, diversity, imine, photocatalysis, visible light

## Abstract

A simple photocatalytic method was developed for the synthesis of unsymmetrical 1,2-diamines by the unprecedented reductive coupling of *N*-benzylidene-[1,1'-biphenyl]-2-amines with an aliphatic amine. The presence of a phenyl substituent in the aniline moiety of the substrate was critical for the reactivity. The reaction proceeded via radical–radical cross-coupling of α-amino radicals generated by proton-coupled single-electron transfer in the presence of an Ir photocatalyst. On the other hand, symmetrical 1,2-diamines were selectively produced from the same starting materials by the judicious choice of the reaction conditions, showcasing the distinct reactivity of *N*-benzylidene-[1,1'-biphenyl]-2-amines. The developed method can be employed for the synthesis of various bulky vicinal diamines, which are potential ligands in stereoselective synthesis.

## Introduction

The selective formation of distinct valuable compounds from the same starting material is a highly attractive divergent approach, though it represents significant synthetic challenges. Recent advances in visible-light photocatalysis, mediated by visible-light-absorbing photosensitizers, have allowed ready access to complex molecules in a controlled manner, where subtle differences in the reaction conditions opened up distinct reaction pathways [1–5].

**Scheme 1:**
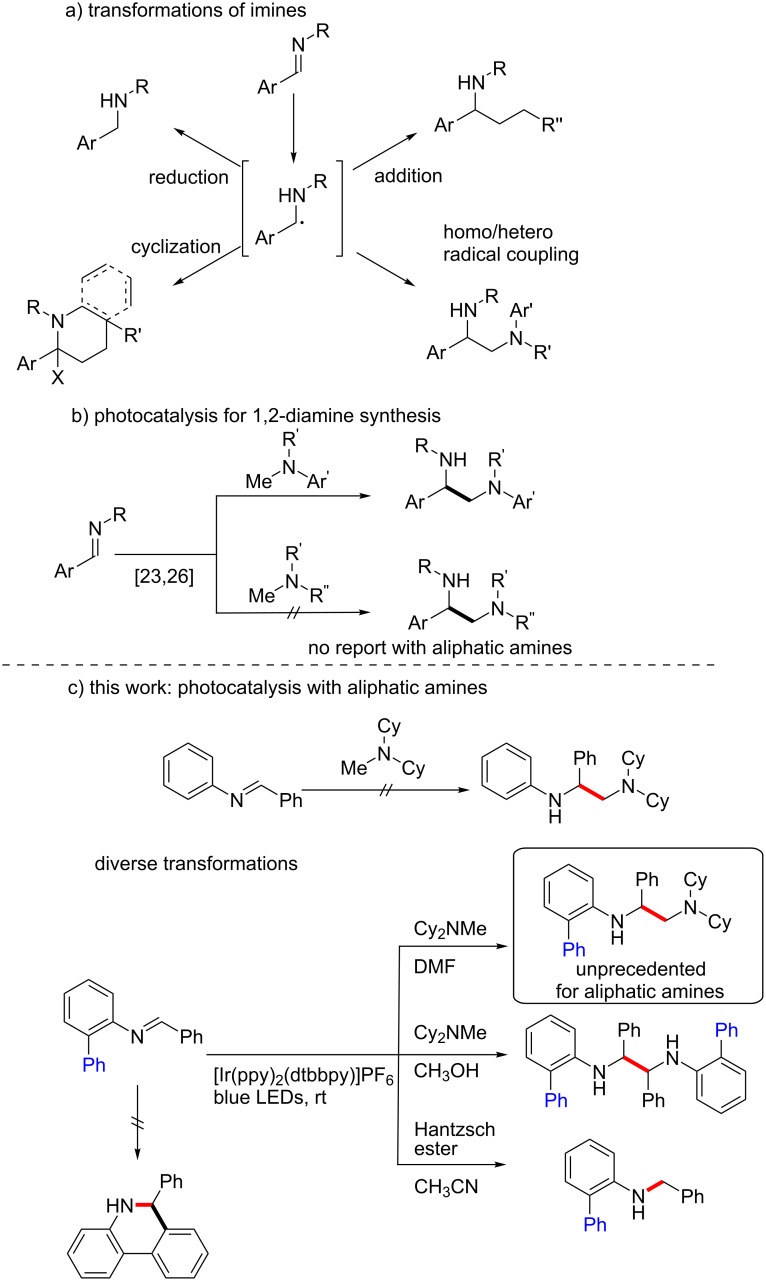
Photocatalytic transformations of imines.

Imines are versatile substrates that can be converted into various azo compounds, depending upon the reaction conditions [6–9]. In particular, the reactivities of *N*-benzylidenes have been extensively explored under visible-light photocatalysis [10–16]. *N*-Benzylidenes can undergo facile single-electron reduction to generate α-amino radical intermediates, which can participate in diverse processes, depending upon the nature of the substrates and the reaction conditions (Scheme 1a). Various amine systems are generated from such intermediates via a wide range of processes, including hydrogen atom abstraction [17–18], the addition to unsaturated compounds [19–21], radical–radical coupling [22–30], and cyclization reactions [31–33]. Among these diverse applications, the Rueping group has reported excellent examples of the reductive umpolung homocoupling of imines and heterocoupling with α-amino radicals for the synthesis of symmetrical and unsymmetrical vicinal diamines (Scheme 1b) [23,26]. Notably, 1,2-diamines have widespread applications as core structures in a variety of natural products, pharmaceuticals, and agrochemicals [34–38] and are valuable ligands [39–40] in stereoselective organic synthesis. Despite the availability of a plethora of synthetic methods for 1,2-diamines [41–45], the reported photocatalytic synthetic methods are mainly limited to aniline-based substrates and do not encompass aliphatic amines.

We planned the synthesis of 1,2-diamine compounds having an aliphatic amine moiety by the intermolecular coupling of *N*-benzylidines with aliphatic amines that not only act as coupling partner but also as electron donors in the photoredox cycle, and the results are reported herein. We began with the reaction of simple *N*-benzylideneaniline. However, this substrate did not furnish the desired products under several different photocatalysis conditions, including those reported by Rueping. We hypothesized that the structural modification of the substrate might affect its reactivity, and to our delight, the introduction of an *ortho*-phenyl moiety on the *N*-benzylideneaniline species provided the desired 1,2-diamine product, wherein the *N*,*N*-dicyclohexylmethylamine (Cy_2_NMe) acted as both the coupling partner and an electron donor in the photoredox cycle (Scheme 1c). It is likely that the presence of the additional phenyl group in the substrate stabilizes the α-amino radical intermediate and modulates its reactivity [46–47]. In addition to the cross-coupled 1,2-diamines, we envisioned the generation of other valuable structural motifs via the careful control of the reaction conditions for the reaction of the biphenyl imine derivative *N*-benzylidene-[1,1'-biphenyl]-2-amine.

## Results and Discussion

With the initial results in hand, we attempted the optimization of the reaction conditions to improve the yield of the 1,2-diamine product (Table 1). Among the series of photocatalysts tested, ranging from the Ru/Ir polypyridyl complexes to organic dyes, [Ir(dtbbpy)(ppy)_2_]PF_6_ was found to be the best and afforded **2a** in 60% yield, along with 11% of the homocoupled product **3a** (Table 1, entries 1–9). The amount of Cy_2_NMe was critical for achieving selectivity, and less than two equivalents of Cy_2_NMe gave greater amounts of the homocoupled product **3a** (Table 1, entry 10). Control experiments showed that the photocatalyst, amine base, and light source are integral aspects of the reaction (Table 1, entries 11–13). DMF was found to be the best solvent and yielded **2a** selectively (Table 1, entries 15–22). On the other hand, the homocoupled **3a** was selectively obtained in protic solvents (Table 1, entries 19 and 22), and in particular, CH_3_OH showed a good reactivity and formed **3a** in 75% yield. Unexpectedly, the cyclized product with the tethered phenyl ring proposed in Scheme 1c was not generated under any of the photocatalytic conditions evaluated.

**Table 1:** Reaction optimization.^a^

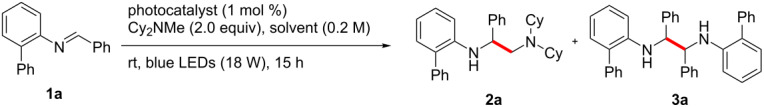					

entry	photocatalyst	variation	solvent	yield (%)^b^	
				**2a**	**3a**

1	[Ru(bpy)_3_]Cl_2_⋅6H_2_O	–	CH_3_CN	12	0
2	[Ru(bpz)_3_](PF_6_)_2_	–	CH_3_CN	0	0
3	Ir(dFppy)_3_	–	CH_3_CN	0	0
4	Ir(ppy)_3_	–	CH_3_CN	3	25
5	[Ir(dF(CF_3_)ppy)_2_(dtbpy)]PF_6_	–	CH_3_CN	38	5
6	[Ir(dtbbpy)(ppy)_2_]PF_6_	–	CH_3_CN	60	11
7	TTPP	–	CH_3_CN	0	0
8	crystal violet	–	CH_3_CN	0	0
9	eosin-Y	–	CH_3_CN	38	0
10	[Ir(dtbbpy)(ppy)_2_]PF_6_	Cy_2_NMe (1 equiv)	CH_3_CN	14	55
11	[Ir(dtbbpy)(ppy)_2_]PF_6_	no Cy_2_NMe	CH_3_CN	0	trace
12	no catalyst	–	CH_3_CN	0	0
13	[Ir(dtbbpy)(ppy)_2_]PF_6_	no light	CH_3_CN	0	0
14	[Ir(dtbbpy)(ppy)_2_]PF_6_	–	DCM	0	16
15	[Ir(dtbbpy)(ppy)_2_]PF_6_	–	DMF	72 (71)	7
16	[Ir(dtbbpy)(ppy)_2_]PF_6_	–	DCE	22	11
17	[Ir(dtbbpy)(ppy)_2_]PF_6_	–	DMSO	37	6
18	[Ir(dtbbpy)(ppy)_2_]PF_6_	–	dioxane	50	13
19	[Ir(dtbbpy)(ppy)_2_]PF_6_	–	TFE	0	20
20	[Ir(dtbbpy)(ppy)_2_]PF_6_	–	acetone	22	trace
21	[Ir(dtbbpy)(ppy)_2_]PF_6_	–	EtOAc	36	8
22	[Ir(dtbbpy)(ppy)_2_]PF_6_	–	CH_3_OH	4	75 (75)
23	[Ir(dtbbpy)(ppy)_2_]PF_6_	–	DMF/CH_3_OH (1:1)	35	29
24	[Ir(dtbbpy)(ppy)_2_]PF_6_	–	DMF/H_2_O (1:1)	25	10
25	[Ir(dtbbpy)(ppy)_2_]PF_6_	–	CH_3_CN/H_2_O (1:1)	15	14

^a^Reaction conditions: **1a** (0.1 mmol), under argon atmosphere. ^b^The yields were determined by ^1^H NMR spectroscopy using 1,3,5-trimethoxybenzene as the internal standard, and the isolated yields are mentioned in parentheses.

**Scheme 2:**
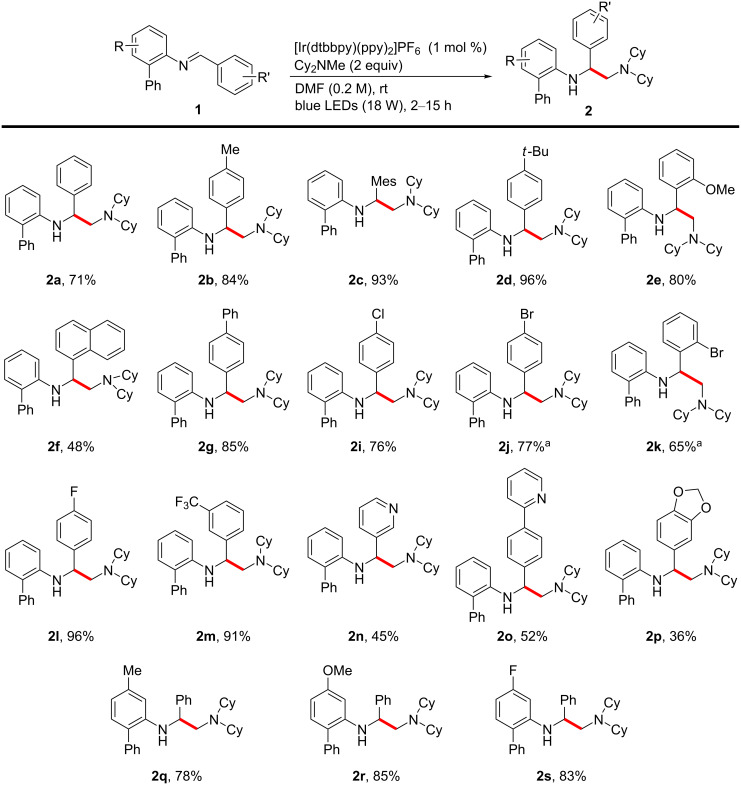
Substrate scope for the radical cross-couplings. Reaction conditions: **1** (0.3 mmol), under argon atmosphere, isolated yields. ^a^The debrominated product was also detected, and the yield represents the mixture of the brominated and debrominated product.

With the optimized conditions in hand, the generality of the transformations was investigated using a wide variety of phenyl-substituted *N*-benzylideneaniline derivatives (Scheme 2). First, the C–C cross-coupling process with Cy_2_NMe was explored, with variations of the benzylidene moiety. The reactions with both electron-donating (**2b**–**2e**) and electron-withdrawing substituents (**2i**–**2m**) proceeded well. Several functional groups, such as benzylic ones (**2b** and **2c**), ethers (**2e**), halogens (**2i** and **2l**) [48–50], the medicinally important CF_3_ group (**2m**), and acetals (**2p**) were tolerated under these mild reaction conditions. The substitution pattern of the aryl groups, such as *ortho* (**2c**, **2e**, and **2k**), *meta* (**2m** and **2p**), and *para* substitution (**2b**–**2d**, **2g**, **2i**, **2l**, **2o**, and **2p**), did not have any significant impact on the reaction outcome. The heteroaryl ring-bearing substrates (**2n** and **2o**) also underwent the transformation, and furnished the valuable vicinal diamine products. The modifications on the aniline moiety were also suitable, and substrates with both electron-donating (**2q** and **2r**) and electron-withdrawing (**2s**) substituents underwent the cross-coupling with excellent reactivities. We also tried the transformation with aliphatic amines other than Cy_2_NMe, such as TMEDA, TEA, and DIPEA. However, these reactions proceeded with a poor chemoselectivity and resulted in the formation of mixtures of several types of amine compounds.

A photochemical quantum yield, determined by using the standard ferrioxalate actinometry, was 83% for the radical cross-coupling between **1a** and Cy_2_NMe (see Supporting Information File 1 for details).

**Scheme 3:**
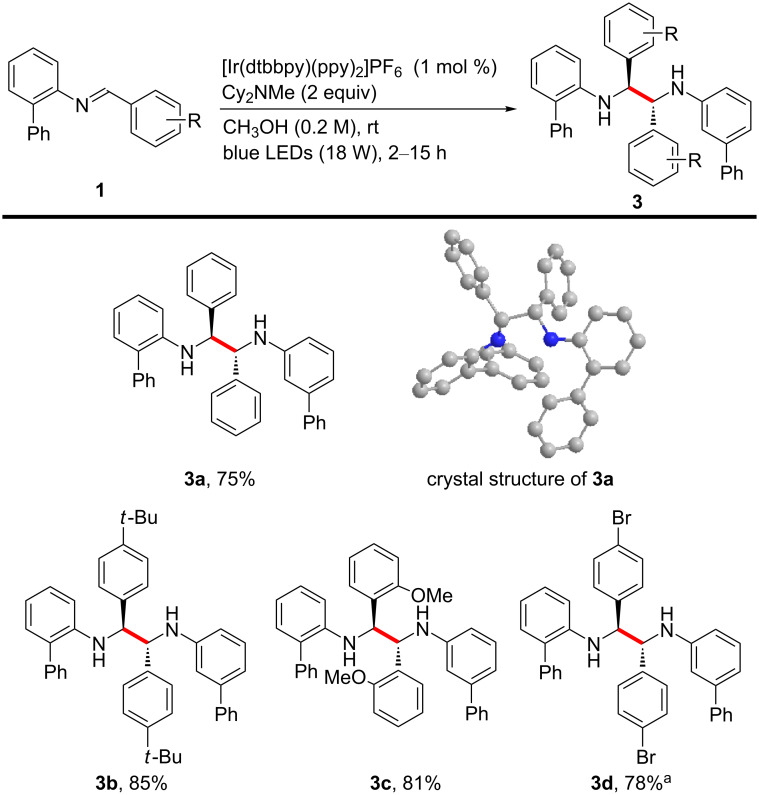
Substrate scope for the homocoupling. Reaction conditions: **1** (0.3 mmol), under argon atmosphere, isolated yield. ^a^The debrominated product was also detected, and the yield represents the mixture of the brominated and debrominated product.

Next, the homocoupling reactions of substituted (*E*)-*N*-benzylidene-[1,1'-biphenyl]-2-amines were studied in CH_3_OH (Scheme 3) [51–56]. It is noteworthy that the resulting symmetrical 1,2-diamine compounds are potential ligands for a variety of organic transformations. The dimerization reactions proceeded well, regardless of the electron density or position of the substituent on the benzylidene moiety. Interestingly, the homocoupling process was highly stereoselective, resulting in the formation of only one diastereomer, probably due to the bulky substitution pattern of the substrates. The structure of the homocoupled 1,2-diamine product **3a** was unambiguously confirmed using X-ray crystallography [57].

**Scheme 4:**
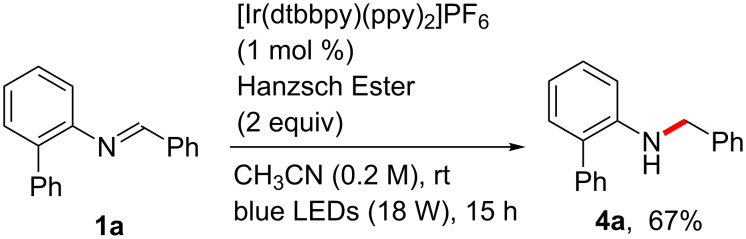
Reduction of the imine **1a** to the amine **4a**.

To further extend the diversity of the reactivity of the imines in this process, we also attempted the use of a reducing agent in the reaction of **1a**. Pleasingly, the reaction of **1a** with 2 equivalents of the Hantzsch ester in CH_3_CN produced the reduced amine product **4a** in 67% yield (Scheme 4) [17–18,58–61].

**Scheme 5:**
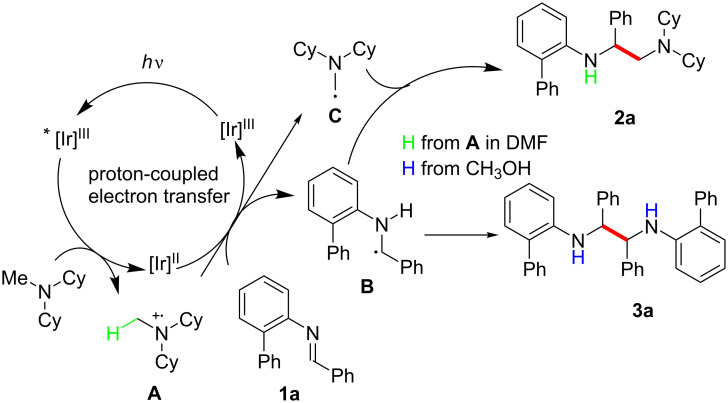
Proposed mechanism.

Based on these observations, a plausible reaction mechanism was proposed for the developed transformation (Scheme 5). Upon visible-light irradiation, the excited photocatalyst [Ir^III^]* is formed and is reductively quenched by single-electron transfer from Cy_2_NMe, resulting in the generation of the highly reducing [Ir^II^] species and the radical cation **A**. To validate the reductive quenching pathway, we carried out Stern−Volmer quenching experiments (Figure S1, Supporting Information File 1). The emission intensity of the excited Ir complex significantly decreased in proportion to the concentration of Cy_2_NMe, while it was much less affected by the concentration of **1a**, confirming the proposed working mode. The formation of **2a** might be attributed to the proton-coupled electron transfer [62–66] from [Ir^II^] to the imine **1a**, where the radical cation **A** donates a proton to **1a** to form the α-amino radical intermediates **B** and **C**, which undergo cross-coupling to give the desired unsymmetrical vicinal diamine **2a**. On the other hand, in CH_3_OH, **1a** preferentially abstracts a proton from CH_3_OH rather than from **A**, which, in turn, prevents the generation of the α-amino radical **C** from Cy_2_NMe, resulting in the homocoupling of **B** to selectively form the symmetrical diamine product **3a**.

## Conclusion

We developed a divergent synthetic approach for the valuable 1,2-diamine motif from *N*-benzylidene-[1,1'-biphenyl]-2-amines by slight alterations to the reaction conditions. The in situ-generated α-amino radical intermediates successfully underwent cross- and homocoupling to yield the unsymmetrical and symmetrical 1,2-diamines, respectively. The presence of the phenyl substituent at the aniline moiety was critical in the heterocoupling with aliphatic amines. Furthermore, the reduced amine product was also obtained by employing the Hantzsch ester. The developed method can be employed for the synthesis of bulky vicinal diamines with potential applications as ligands for stereoselective synthesis.

## Experimental

An oven-dried resealable tube, equipped with a magnetic stir bar, was charged with the *N*-benzylidene-[1,1'-biphenyl]-2-amine derivative (0.3 mmol), [Ir(dtbbpy)(ppy)_2_]PF_6_ (0.003 mmol), and Cy_2_NMe (0.6 mmol). The reaction mixture was purged with argon for 20 min. Then, degassed DMF or CH_3_OH was added to the reaction mixture under inert conditions. The reaction mixture was stirred at ambient temperature for 2**–**15 h under visible-light irradiation with blue LEDs (18 W). The progress of the reaction was monitored by using TLC. Upon the completion of the reaction, the crude product was diluted with ethyl acetate and washed with brine. The organic layer was dried over anhydrous MgSO_4_ and concentrated in vacuo. The desired vicinal diamine product was puriﬁed by silica-gel column chromatography using hexane/EtOAc as the eluent.

## Supporting Information

File 1 Additional experimental details and analytical data.

File 2 Crystal data for **3a**.
