# Supplementary material for: Distinctive reactivity of N-benzylidene-[1,1'-biphenyl]-2-amines under photoredox conditions
Source: Beilstein J Org Chem. 2020 Jun 18;16:1335–42. doi: 10.3762/bjoc.16.114 (PMC7308614; doi:10.3762/bjoc.16.114)

BEILSTEIN JOURNAL OF ORGANIC CHEMISTRY

Supporting Information

for

Distinctive reactivity of *N*-benzylidene-[1,1'-biphenyl]-2-amines under photoredox conditions

Shrikant D. Tambe, Kwan Hong Min, Naeem Iqbal and Eun Jin Cho

Beilstein J. Org. Chem. **2020,** *16,* 1335–1342. doi:10.3762/bjoc.16.114

Additional experimental details and analytical data

License and Terms: This is a supporting information file under the terms of the Creative Commons Attribution License (http://creativecommons.org/licenses/by/4.0). Please note that the reuse, redistribution and reproduction in particular requires that the authors and source are credited. The license is subject to the *Beilstein Journal of Organic Chemistry* terms and conditions: (https://www.beilstein-journals.org/bjoc)

**General considerations**	S2
**Experimental details**	S2
**Additional experiments**	S4
**References**	S7
**Analytic data for the synthesized compounds**	S7
**NMR spectra**	S17

General considerations

General reagent information

All other reagents required for the syntheses of the diamines **2** and **3** were purchased from Sigma-Aldrich, Alfa Aesar, TCI, or Acros Organics. Flash column chromatography was performed using Zeochem silica gel 60 (60−200 mesh).

General analytical information

The synthesized diamines **2** and **3** were characterized using ^{1}H NMR, ^{13}C NMR, and FTIR spectroscopy. NMR spectra were recorded on a Varian 600 MHz instrument (600 MHz for ^{1}H NMR and 151 MHz for ^{13}C NMR). Copies of the ^{1}H and ^{13}C NMR spectra are included at the end of the Supporting Information. ^{1}H NMR chemical shifts are reported in parts per million (ppm) relative to residual chloroform (7.26 ppm) in the deuterated solvent. ^{13}C{^{1}H} NMR spectra are reported in ppm relative to deuterochloroform (77.23 ppm). Coupling constants were reported in Hz. FTIR spectra were recorded on a Nicolet 6700 FT-IR spectrometer (ThermoFisher). Mass spectral data of all unknown compounds were acquired at the Korea Basic Science Institute (Daegu) on a Jeol JMS 700 high-resolution mass spectrometer. A quadrupole mass analyzer was used for HRMS measurements. Melting points were recorded on a Stuart SMP30 apparatus.

Experimental details

General experimental procedure for the synthesis of the *N*-benzylidene-[1,1'-biphenyl]-2-amines 1[S1]

An oven-dried 100-mL round-bottom flask equipped with a magnetic stir bar was charged with the [1,1'-biphenyl]-2-amine derivative (5 mmol), the benzaldehyde derivative (5 mmol), and anhydrous $MgSO_4$ (5 mmol) in dichloromethane (7.5 mL). Then, the reaction mixture was stirred for 15 h at room temperature, and the progress of the reaction was monitored by TLC. After completion of reaction, $MgSO_4$ was removed by filtration, and the filtrate was concentrated in vacuo to get the corresponding imine **1**, which was utilized for the coupling reaction without further purification.

General experimental procedure for the synthesis of the 1,2-diamines 2 and 3

An oven-dried resealable tube, equipped with a magnetic stir bar, was charged with the *N*-benzylidene-[1,1'-biphenyl]-2-amine derivative (0.3 mmol), [Ir(dtbbpy)(ppy)$_2$]PF$_6$ (0.003 mmol), and Cy$_2$NMe (0.6 mmol). The reaction mixture was purged with argon for 20 min. Then, degassed DMF or CH_3OH was added to the reaction mixture under inert conditions. The reaction mixture was stirred at ambient temperature for 2–15 h under visible-light irradiation with blue LEDs (18 W). The progress of the reaction was monitored by using TLC. Upon reaction completion, the crude product was diluted with ethyl acetate and washed with brine. The organic layer was dried over anhydrous $MgSO_4$ and concentrated in vacuo. The desired vicinal diamine products were purified by silica-gel column chromatography using hexane/EtOAc as the eluent.

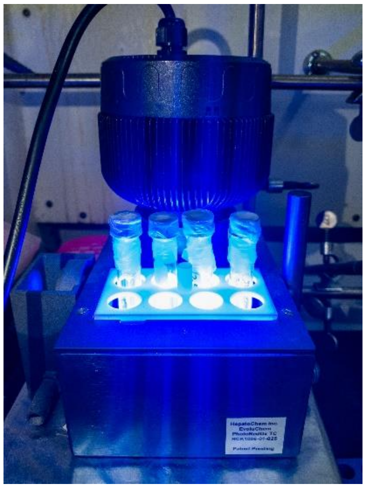

The photoreactions were performed using a 18 W blue LED (450 nm) and a PhotoRedOx Box® supplied by HepatoChem.

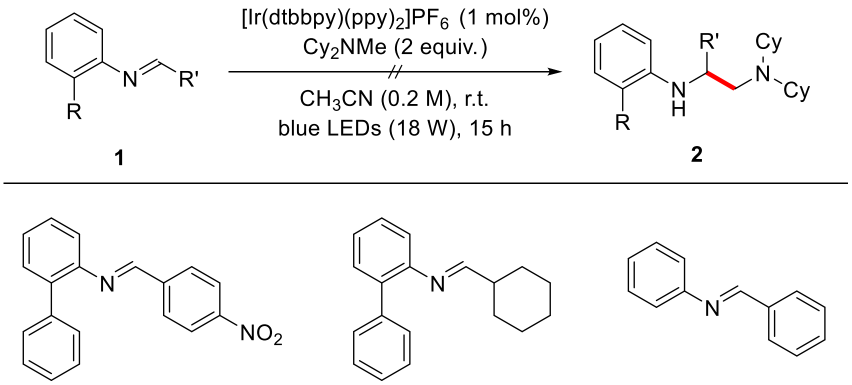

Scheme S1. Unsuccessful substrates for the radical cross-coupling.

Additional experiments

The photoluminescence quenching experiment

Photoluminescence quenching experiments of the photoexcited $[Ir(dtbbpy)_2(ppy)]PF_6$ were performed using **1a** and Cy_2NMe. The Stern–Volmer constants K_{SV} (**1a**) = 33.7 M^{-1} and K_{SV} (Cy_2NMe) = 88.5 M^{-1} were obtained, supporting the proposed reductive quenching pathway.

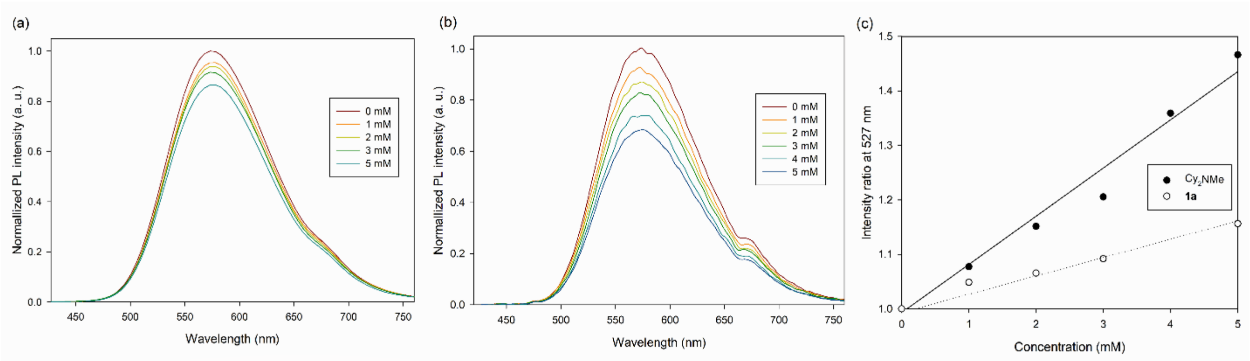

Figure S1. (a) Photoluminescence spectra (excitation wavelength = 417 nm) of 100 μM $[Ir(dtbbpy)_2(ppy)]PF_6$ (Ar-saturated DMF) obtained with increasing the concentration of **1a** (0–5 mM). (b) Photoluminescence spectra (excitation wavelength = 417 nm) of 100 μM $[Ir(dtbbpy)_2(ppy)]PF_6$ (Ar-saturated DMF) obtained with increasing the concentration of Cy_2NMe (0–5 mM). (c) Stern–Volmer plots.

Determination of the quantum yield[S2]

Determination of light intensity at 450 nm:

The photon flux of the spectrophotometer was determined by standard ferrioxalate actinometry. A 0.15 M solution of $K_3[Fe(C_2O_4)_3]$ was prepared by dissolving 0.74 g of potassium ferrioxalate hydrate in 10 mL of 0.05 M H_2SO_4. A buffered solution of phenanthroline was prepared by dissolving 10 mg of phenanthroline and 2.25 g of sodium acetate in 10 mL of 0.5 M H_2SO_4. Both solutions were stored in the dark. To determine the photon flux of the spectrophotometer, 2.0 mL of the ferrioxalate solution was placed in a cuvette and irradiated for 90.0 seconds at λ = 450 nm with an emission slit width at 10.0 nm. After irradiation, 0.35 mL of the phenanthroline solution was added to the cuvette. The solution was then allowed to rest for 1 h to allow the ferrous ions to completely coordinate to the phenanthroline. The absorbance of the solution was measured at 510 nm. A nonirradiated sample was also prepared and the absorbance at 510 nm measured.

Conversion of Fe^{3+} to Fe^{2+} was calculated using Eq. 1.

$$mol\ Fe^{2+} = \frac{V \cdot \Delta A}{l \cdot \varepsilon} \qquad \text{(Eq. 1)}$$

Where *V* is the total volume (0.015 L) of the solution after the addition of phenanthroline, Δ*A* is the difference in absorbance at 510 nm between the irradiated and nonirradiated solutions, *l* is the path length (1.000 cm), and ε is the molar absorptivity at 510 nm (11,100 $L{\cdot}mol^{-1}{\cdot}cm^{-1}$).

The photon flux can be calculated using Eq. 2.

$$photo\ flux = \frac{mol\ Fe^{2+}}{\Phi \cdot t \cdot f} \qquad \text{(Eq. 2)}$$

Where Φ is the quantum yield for the ferrioxalate actinometer (1.01 for a 0.15 M solution at λ = 450 nm), *t* is the time (90.0 s), and *f* is the fraction of light absorbed at λ = 450 nm (0.99833). The photon flux was calculated to be 1.9×10^{-8} einstein s^{-1}.

Sample calculation;

$$mol\ Fe^{2+} = \frac{0.015\ \mathrm{L} \bullet 1.3051}{1.000\ \mathrm{cm} \bullet 11{,}100 L^{-1}\ \mathrm{mol}\ cm^{-1}} = 1.763 \times 10^{-6}\ \mathrm{mol}^{-1}$$

$$\text{photon flux} = \frac{1.763 \times 10^{-6}\ \mathrm{mol}}{1.01 \bullet 90.0\ \mathrm{s} \bullet 0.99833} = 1.9 \times 10^{-8}\ \text{einstein s}^{-1}$$

Determination of the quantum yield:

Radical cross-coupling reaction.

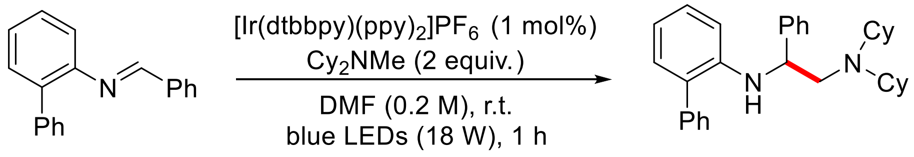

1a **2a**

A cuvette was charged with **1a** (0.1 mmol, 1 equiv.), Cy_2NMe (0.2 mmol, 2 equiv.), $[Ir(dtbbpy)(ppy)_2]PF_6$ (0.001 mmol, 1 mol %), and 0.5 mL DMF (0.2 M). The cuvette was then capped with a PTFE stopper. The sample was stirred and irradiated (λ = 450 nm, slit width = 10.0 nm) for 3600 s (60 min). After irradiation, the solution was passed through a silica plug. The yield of the product was determined by ^{1}H NMR using 1,3,5-trimethoxybenzene as an internal standard. The quantum yield was determined using Eq. 3. Essentially, all incident light (f > 0.999) is absorbed by $[Ir(dtbbpy)(ppy)_2]PF_6$ at the reaction conditions described above.

$$\Phi = \frac{mol\ of\ product}{flux \cdot t \cdot f} \qquad \text{(Eq. 3)}$$

Experiment 1: 25.7 mg (0.1 mmol) [1,1'-biphenyl]-2-amine, 42 μL (0.2 mmol) Cy_2NMe, 0.9 mg (0.001 mmol) $[Ir(dtbbpy)(ppy)_2]PF_6$, and 0.5 mL (0.2 M) DMF, after 3600 s, yielded 57% of **2a**, Φ(57%) = 0.83.

Sample quantum yield calculation:

$$\Phi = \frac{5.7 \times 10^{-5}\ \text{mol}}{1.9 \times 10^{-8}\text{einstein}\ s^{-1} \bullet 3600\ \text{s} \bullet 0.99833} = 0.83$$

Reference

S1. Shiino, M.; Watanabe, Y.; Umezawa, K.; *Bioorg. Med. Chem.*, **2001**, *9*, 1233.

S2. Cismesia, M. A.; Yoon T. P.; *Chem. Sci.,* **2015**, *6*, 5426

Analytic data for the synthesized compounds

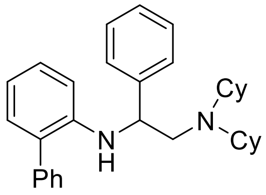

N[1]-([1,1'-Biphenyl]-2-yl)-*N*[2],*N*[2]-dicyclohexyl-1-phenylethane-1,2-diamine (**2a**): white solid (71%); mp 132-137 °C; **^{1}H NMR (600 MHz, $CDCl_3$)** δ 7.56 (d, *J* = 7.6 Hz, 2H), 7.48 – 7.44 (m, 4H), 7.40 – 7.34 (m, 3H), 7.28 (t, *J* = 7.3 Hz, 1H), 7.10 (d, *J* = 7.3 Hz, 1H), 7.02 (dd, *J* = 8.2, 7.4 Hz, 1H), 6.73 (dd, *J* = 7.4, 7.3 Hz, 1H), 6.31 (d, *J* = 8.2 Hz, 1H), 5.38 (s, 1H), 4.11 (dd, *J* = 10.9, 4.3 Hz, 1H), 2.91 (dd, *J* = 13.7, 4.3 Hz, 1H), 2.38 (dd, *J* = 13.7, 10.9 Hz, 1H), 2.35 – 2.29 (m, 2H), 1.69 – 1.63 (m, 4H), 1.60 – 1.53 (m, 4H), 1.14 – 1.06 (m, 8H), 1.02 – 0.94 (m, 4H); **^{13}C NMR (151 MHz, $CDCl_3$)** δ 145.79, 144.24, 140.33, 130.06, 130.04, 129.06, 128.83, 128.75, 128.38, 127.06, 126.45, 116.85, 112.75, 57.80, 57.19, 54.01, 32.70, 31.03, 26.68, 26.65, 26.28; **IR (neat)**: ν_{max} = 3305, 3026, 2927, 2851, 1603, 1505, 1488, 749, 700 cm^{-1}; **HRMS** m/z (FAB) calc. for $C_{32}H_{41}N_2$ [M+H] =453.3270, Found 453.3267; ***R_f*** 0.61 (hex/EtOAc, 9/1)

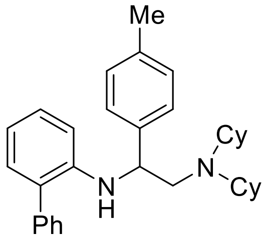

N[1]-([1,1'-Biphenyl]-2-yl)-*N*[2],*N*[2]-dicyclohexyl-1-(*p*-tolyl)ethane-1,2-diamine (**2b**): white solid (84%); mp 140-145 °C; **^{1}H NMR (600 MHz, $CDCl_3$)** δ 7.55 (d, *J* = 7.7 Hz, 2H), 7.45 (dd, *J* = 7.3, 6.7 Hz, 2H), 7.36 – 7.32 (m, 3H), 7.18 (d, *J* = 7.3 Hz, 2H), 7.09 (d, *J* = 7.4 Hz, 1H), 7.02 (dd, *J* = 8.2, 7.3 Hz, 1H), 6.71 (dd, *J* = 7.4, 7.3 Hz, 1H), 6.32 (d, *J* = 8.2 Hz, 1H), 5.36 (s, 1H), 4.07 (dd, *J* = 10.7, 3.1 Hz, 2H), 2.88 (dd, *J* = 13.7, 3.1 Hz, 1H), 2.38 (s, 3H), 2.34 (dd, *J* = 13.7, 10.7

Hz, 1H), 2.33 – 2.28 (m, 2H), 1.69 – 1.62 (m, 4H), 1.59 – 1.52 (m, 4H), 1.14 – 1.05 (m, 8H), 1.01 – 0.93 (m, 4H); **^{13}C NMR (151 MHz, CDCl$_3$)** δ 145.91, 141.21, 140.36, 136.57, 130.05, 130.03, 129.55, 129.03, 128.73, 128.38, 127.03, 126.35, 116.78, 112.76, 57.78, 56.90, 54.10, 32.72, 30.99, 26.69, 26.65, 26.29, 21.36; **IR (neat)**: v_{max} = 3303, 3018, 2927, 2851, 1603, 1507, 1488, 735, 701 cm^{-1}; **HRMS** m/z (FAB) calc. for $C_{33}H_{43}N_2$ [M+H] = 467.3426, Found 467.3425; ***R_f*** 0.63 (hex/EtOAc, 9/1)

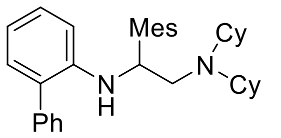

N-(3-([1,1'-Biphenyl]-2-yl)-2-mesitylpropyl)-*N*-cyclohexylcyclohexanamine (**2c**): white solid (93%); mp 147-152 °C; **^{1}H NMR (600 MHz, CDCl$_3$)** δ 7.45 (dd, *J* = 7.8, 1.7 Hz, 2H), 7.42 (dd, *J* = 7.8, 7.2 Hz, 2H), 7.33 (t, *J* = 7.2 Hz, 1H), 7.06 (dd, *J* = 7.3, 1.4 Hz, 1H), 7.00 (ddd, *J* = 8.2, 7.3, 1.6 Hz, 1H), 6.90 (s, 1H), 6.79 (s, 1H), 6.67 (ddd, *J* = 7.3, 7.3, 1.4 Hz, 1H), 6.20 (d, *J* = 8.2 Hz, 1H), 5.23 (s, 1H), 4.54 (dd, *J* = 11.6, 4.5 Hz, 1H), 2.76 (dd, *J* = 13.7, 4.5 Hz, 1H), 2.65 (dd, *J* = 13.7, 11.6 Hz, 1H), 2.49 (s, 3H), 2.46 (s, 3H), 2.35 – 2.30 (m, 2H), 2.28 (s, 3H), 1.69 – 1.60 (m, 6H), 1.57 – 1.53 (m, 2H), 1.15 – 1.05 (m, 6H), 1.02 – 0.94 (m, 6H); **^{13}C NMR (151 MHz, CDCl$_3$)** δ 146.29, 140.35, 137.12, 136.02, 135.38, 135.20, 131.94, 130.10, 130.03, 129.85, 128.74, 128.63, 127.02, 116.20, 110.97, 57.38, 53.01, 48.26, 33.23, 30.80, 26.78, 26.70, 26.37, 21.62, 21.07, 20.96; **IR (neat)**: v_{max} = 3309, 2987, 2925, 2852, 1604, 1506, 1489, 733, 701 cm^{-1}; ***R_f*** 0.62 (hex/EtOAc, 9/1)

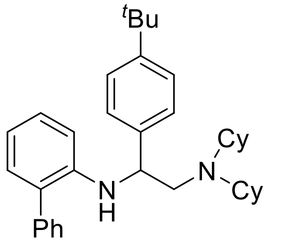

*N*1-([1,1'-Biphenyl]-2-yl)-1-(4-(*tert*-butyl)phenyl)-*N^2*,*N^2*-dicyclohexylethane-1,2-diamine (**2d**): white solid (96%); mp 157-162 °C; **^{1}H NMR (600 MHz, CDCl$_3$)** δ 7.55 (d, *J* = 7.4 Hz, 2H), 7.45 (t, *J* = 7.6 Hz, 2H), 7.39 – 7.32 (m, 5H), 7.08 (dd, *J* = 7.3, 1.3 Hz, 1H), 7.02 (ddd, *J* = 7.9, 7.5 1.3 Hz, 1H), 6.71 (dd, *J* = 7.5, 7.3 Hz, 1H), 6.33 (d, *J* = 7.9 Hz, 1H), 5.35 (s, 1H), 4.08 (dd, *J* = 10.7, 4.4 Hz, 1H), 2.89 (dd, *J* = 13.6, 4.4 Hz, 1H), 2.38 (dd, *J* = 13.6, 10.7 Hz, 1H), 2.33 – 2.28 (m, 2H), 1.68 – 1.62 (m, 4H), 1.57 – 1.51 (m, 4H), 1.35 (s, 8H), 1.13 – 1.05 (m, 8H), 1.01 – 0.93 (m, 4H); **^{13}C NMR (151 MHz, CDCl$_3$)** δ 149.75, 145.93, 141.05, 140.39, 130.04, 130.01, 129.00, 128.73, 128.37, 127.02, 126.05, 125.67, 116.72, 112.71, 57.77, 56.76, 53.99, 34.66, 32.69, 31.67, 31.06, 26.69, 26.66, 26.29; **IR (neat)**: v_{max} = 3307, 3018, 2928, 2852, 1604, 1506, 1488, 746, 701 cm^{-1}; **HRMS** m/z (FAB) calc. for $C_{36}H_{49}N_2$ [M+H] = 509.3896, Found 509.3893; ***R_f*** 0.66 (hex/EtOAc, 9/1)

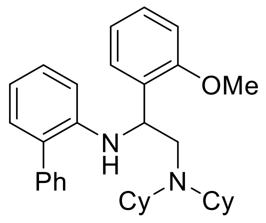

N[1]-([1,1'-Biphenyl]-2-yl)-*N*[2],*N*[2]-dicyclohexyl-1-(2-methoxyphenyl)ethane-1,2-diamine (**2e**): white solid (80%); mp 155-160 °C; **^{1}H NMR (600 MHz, $CDCl_3$)** δ 7.54 (d, *J* = 7.6 Hz, 2H), 7.44 (dd, *J* = 7.6, 7.3 Hz, 2H), 7.34 (t, *J* = 7.3 Hz, 1H), 7.29 (dd, *J* = 8.1, 7.5 Hz, 1H), 7.08 (d, *J* = 7.3 Hz, 1H), 7.05 (d, *J* = 7.5 Hz, 1H), 7.03 – 7.00 (m, 2H), 6.81 (d, *J* = 8.1 Hz, 1H), 6.72 (dd, *J* = 7.4, 7.3 Hz, 1H), 6.33 (d, *J* = 8.2 Hz, 1H), 5.35 (s, 1H), 4.06 (dd, *J* = 11.5, 4.5 Hz, 1H), 3.84 (s, 3H), 2.90 (dd, *J* = 12.8, 4.5 Hz, 1H), 2.37 (dd, *J* = 12.8, 11.5 Hz, 1H), 2.33 – 2.27 (m, 2H), 1.68 – 1.62 (m, 4H), 1.57 – 1.51 (m, 4H), 1.13 – 1.04 (m, 8H), 1.00 – 0.93 (m, 4H); **^{13}C NMR (151 MHz, $CDCl_3$)** δ 159.95, 146.06, 145.60, 140.10, 129.82, 129.80, 129.62, 128.84, 128.52, 128.18, 126.84, 118.66, 116.68, 112.55, 112.29, 111.60, 57.58, 57.05, 55.18, 53.67, 32.48, 30.79, 26.46, 26.42, 26.05; **IR (neat)**: ν_{max} = 3301, 3056, 2926, 2851, 1597, 1505, 1487, 1270, 1047, 738, 700 cm^{-1}; **HRMS** m/z (FAB) calc. for $C_{33}H_{43}N_2O$ [M+H] = 483.3375, Found 483.3373; ***R_f*** 0.54 (hex/EtOAc, 9/1)

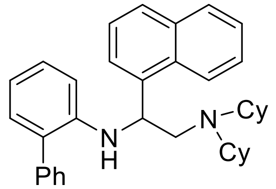

N-([1,1'-Biphenyl]-2-yl)-*N*',*N*'-dicyclohexyl-1-(naphthalen-1-yl)methanediamine (**2f**): white solid (48%); mp 153-158 °C; **^{1}H NMR (600 MHz, $CDCl_3$)** δ 7.91 – 7.84 (m, 4H), 7.62 – 7.57 (m, 3H), 7.51 – 7.45 (m, 4H), 7.37 (td, *J* = 7.32, 1.16 Hz, 1H), 7.11 (d, *J* = 7.5 Hz, 1H), 6.95 (t, *J* = 8.3, 7.3 Hz, 1H), 6.71 (t, *J* = 7.5, 7.3 Hz, 1H), 6.36 (d, *J* = 8.3 Hz, 1H), 5.48 (s, 1H), 4.27 (dd, *J* = 10.6, 4.2 Hz, 1H), 2.97 (dd, *J* = 13.3, 4.2 Hz, 1H), 2.44 (dd, *J* = 13.3, 10.6 Hz, 1H), 2.38 – 2.32 (m, 2H), 1.69 – 1.63 (m, 4H), 1.60 – 1.51 (m, 4H), 1.15 – 1.06 (m, 8H), 1.01 – 0.93 (m, 4H); **^{13}C NMR (151 MHz, $CDCl_3$)** δ 145.90, 142.01, 140.34, 133.93, 133.15, 130.09, 129.13, 129.11, 128.79, 128.65, 128.43, 127.97, 127.95, 127.12, 126.12, 125.57, 124.99, 124.87, 116.97, 112.94, 57.86, 57.51, 53.97, 32.75, 31.01, 26.70, 26.66, 26.29; **IR (neat)**: ν_{max} = 3296, 3055, 2928, 2852, 1602, 1506, 1488, 744, 701 cm^{-1}; **HRMS** m/z (FAB) calc. for $C_{36}H_{43}N_2$ [M+H] = 503.3426, Found 503.3422; ***R_f*** 0.61 (hex/EtOAc, 9/1)

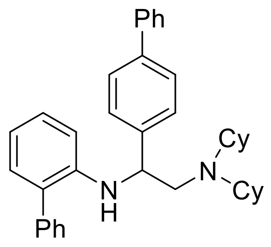

N[1]-([1,1'-Biphenyl]-2-yl)-1-([1,1'-biphenyl]-4-yl)-*N*[2],*N*[2]-dicyclohexylethane - 1,2-diamine (**2g**): white solid (85%); mp 142-147 °C; **^{1}H NMR (600 MHz, $CDCl_3$)** δ 7.60 (d, *J* = 6.9 Hz, 2H), 7.58 (d, *J* = 8.2 Hz, 2H), 7.54 (d, *J* = 6.7 Hz, 2H), 7.49 (d, *J* = 8.2 Hz, 2H), 7.46 – 7.42 (m, 4H), 7.33 (t, *J* = 7.4 Hz, 2H), 7.08 (dd, *J* = 7.4, 1.7 Hz, 1H), 7.02 (ddd, *J* = 8.2, 7.3, 1.7 Hz, 1H), 6.71 (ddd, *J* = 7.4, 7.3, 1.1 Hz, 1H), 6.33 (dd, *J* = 8.2, 1.1 Hz, 1H), 5.38 (s, 1H), 4.12 (dd, *J* = 11.0, 4.5 Hz, 1H), 2.92 (dd, *J* = 13.6, 4.5 Hz, 1H), 2.39 (dd, *J* = 13.6, 11.0 Hz, 1H), 2.34 – 2.28 (m, 2H), 1.67 – 1.62 (m, 4H), 1.57 – 1.52 (m, 4H), 1.00 – 0.93 (m, 8H), 1.00 – 0.93 (m, 4H); **^{13}C NMR (151 MHz, $CDCl_3$)** δ 145.82, 143.40, 141.33, 140.33, 140.03, 130.12, 130.07, 129.13, 128.93, 128.79, 128.43, 127.62, 127.28, 127.11, 126.90, 124.01, 116.96, 112.81, 57.85, 56.98, 54.00, 32.72, 31.06, 26.69, 26.66, 26.28; **IR (neat)**: $ν_{max}$ = 3297, 2987, 2927, 2853, 1602, 1505, 749, 699 cm^{-1}; **HRMS** m/z (FAB) calc. for $C_{38}H_{45}N_2$ [M+H] = 529.3583, Found 529.3586; ***R_f*** 0.52 (hex/EtOAc, 9/1)

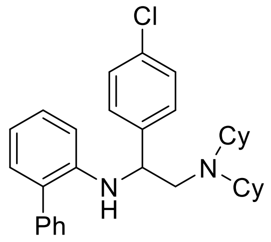

N[1]-([1,1'-Biphenyl]-2-yl)-1-(4-chlorophenyl)-*N*[2],*N*[2]-dicyclohexylethane-1,2-diamine (**2i**): white solid (76%); mp 80-85 °C; **^{1}H NMR (600 MHz, $CDCl_3$)** δ 7.55 (d, *J* = 7.6 Hz, 2H), 7.46 (dd, *J* = 7.6, 7.4 Hz, 2H), 7.39 (d, *J* = 8.1 Hz, 2H), 7.38 – 7.33 (m, 3H), 7.11 (d, *J* = 7.4 Hz, 1H), 7.04 (dd, *J* = 8.2, 7.4 Hz, 1H), 6.75 (dd, *J* = 7.4, 7.4 Hz, 1H), 6.27 (d, *J* = 8.2 Hz, 1H), 5.38 (s, 1H), 4.09 (dd, *J* = 10.9, 4.4 Hz, 1H), 2.88 (dd, *J* = 13.6, 4.4 Hz, 1H), 2.35 (d, *J* = 13.6, 10.9 Hz, 1H), 2.34 – 2.29 (m, 2H), 1.70 – 1.64 (m, 4H), 1.59 – 1.54 (m, 4H), 1.14 – 1.06 (m, 8H), 1.01 – 0.94 (m, 4H); **^{13}C NMR (151 MHz, $CDCl_3$)** δ 145.49, 142.79, 140.16, 132.59, 130.16, 130.00, 129.20, 129.01, 128.77, 128.38, 127.82, 127.14, 117.17, 112.68, 57.82, 56.71, 53.88, 32.67, 31.00, 26.64, 26.61, 26.24; **IR (neat)**: $ν_{max}$ = 3301, 3020, 2927, 2852, 1604, 1505, 1485, 747, 739, 701 cm^{-1}; **HRMS** m/z (FAB) calc. for $C_{32}H_{40}ClN_2$ [M+H] = 487.2880, Found 487.2881; ***R_f*** 0.66 (hex/EtOAc, 9/1)

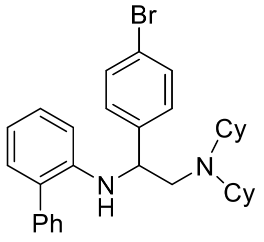

N^1-([1,1'-Biphenyl]-2-yl)-1-(4-bromophenyl)-*N^2*,*N^2*-dicyclohexylethane-1,2-diamine (**2j**): pale yellow solid (77%); mp 138-142 °C; **^{1}H NMR (600 MHz, $CDCl_3$)** δ 7.50 (d, *J* = 6.9 Hz, 2H), 7.46 (d, *J* = 8.4 Hz, 2H), 7.43 (dd, *J* = 7.6, 7.3 Hz, 2H), 7.33 (t, *J* = 7.4 Hz, 1H), 7.30 (d, *J* = 8.4 Hz, 2H), 7.07 (dd, *J* = 7.4, 1.7 Hz, 1H), 7.00 (dd, *J* = 8.1, 7.5 Hz, 1H), 6.72 (dd, *J* = 7.5, 7.4 Hz, 1H), 6.22 (d, *J* = 8.1 Hz, 1H), 5.33 (s, 1H), 4.03 (dd, *J* = 10.6, 4.4 Hz, 1H), 2.84 (dd, *J* = 13.6, 4.4 Hz, 2H), 2.29 (dd, *J* = 13.6, 10.6 Hz, 2H), 2.31 – 2.25 (m, 2H), 1.65 – 1.60 (m, 4H), 1.54 – 1.50 (m, 4H), 1.09 – 1.01 (m, 8H), 0.97 – 0.90 (m, 4H); **^{13}C NMR (151 MHz, $CDCl_3$)** δ 145.49, 143.38, 131.97, 130.19, 130.01, 129.22, 128.79, 128.40, 128.25, 127.16, 120.67, 117.20, 112.70, 57.82, 56.77, 53.83, 32.68, 31.00, 26.65, 26.61, 26.24; **IR (neat)**: ν_{max} = 3301, 3059, 2927, 2852, 1603, 1506, 1487, 736, 701, 532 cm^{-1}; ***R_f*** 0.63 (hex/EtOAc, 9/1)

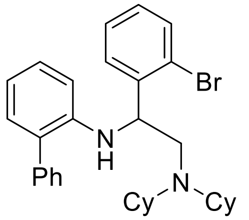

N^1-([1,1'-Biphenyl]-2-yl)-1-(2-bromophenyl)-*N^2*,*N^2*-dicyclohexylethane-1,2-diamine (**2k**): white solid (65%); mp 142-147 °C; **^{1}H NMR (600 MHz, $CDCl_3$)** δ 7.53 (d, *J* = 7.2 Hz, 2H), 7.47 – 7.42 (m, 5H), 7.37 – 7.33 (m, 3H), 7.27 – 7.24 (m, 1H), 7.07 (dd, *J* = 7.4, 1.6 Hz, 1H), 6.99 (ddd, *J* = 7.9, 7.4, 1.6 Hz, 1H), 6.70 (ddd, *J* = 7.4, 7.4, 1.1 Hz, 1H), 6.28 (d, *J* = 7.9 Hz, 1H), 5.35 (s, 1H), 4.08 (dd, *J* = 10.8, 4.4 Hz, 1H), 2.88 (dd, *J* = 13.6, 4.4 Hz, 1H), 2.35 (dd, *J* = 13.6, 10.8 Hz, 1H), 2.32 – 2.27 (m, 2H), 1.66 – 1.61 (m, 4H), 1.56 – 1.50 (m, 4H), 1.11 – 1.04 (m, 8H), 0.99 – 0.92 (m, 4H); **^{13}C NMR (151 MHz, $CDCl_3$)** δ 145.80, 144.25, 140.33, 130.07, 130.05, 129.07, 128.84, 128.75, 128.38, 127.07, 126.46, 116.85, 112.75, 57.80, 57.19, 54.01, 32.70, 31.03, 26.68, 26.65, 26.28; **IR (neat)**: ν_{max} = 3309, 3060, 2928, 2852, 1603, 1505, 1488, 747, 700, 661 cm^{-1}; ***R_f*** 0.63 (hex/EtOAc, 9/1)

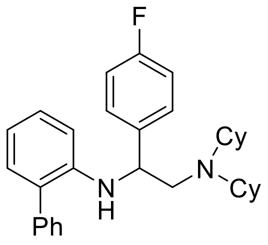

N^1-([1,1'-Biphenyl]-2-yl)-*N^2*,*N^2*-dicyclohexyl-1-(4-fluorophenyl)ethane-1,2-diamine (**2l**): white solid (96%); mp 145-150 °C; **^{1}H NMR (600 MHz, $CDCl_3$)** δ 7.53 (d, *J* = 7.5 Hz, 2H), 7.45 (dd, *J* = 7.5, 7.2 Hz, 2H), 7.40 (dd, *J* = 8.5, $^4J_{H\text{-}F}$ = 5.3 Hz, 2H), 7.35 (t, *J* = 7.2 Hz, 1H), 7.10 (d, *J* = 7.4 Hz, 1H), 7.05 (dd, $J_{H\text{-}F}$ = 8.9 Hz, *J* = 8.5 Hz, 2H), 7.02 (dd, *J* = 7.9, 6.8 Hz, 1H), 6.73 (dd, *J* = 7.4, 6.8 Hz, 1H), 6.27 (d, *J* = 7.9 Hz, 1H), 5.36 (s, 1H), 4.08 (dd, *J* = 10.8, 4.7 Hz, 2H), 2.87 (dd, *J* = 13.5, 4.5 Hz, 1H), 2.34 (dd, 1H), 2.32 – 2.27 (m, 4H), 1.68 – 1.63 (m, 7H), 1.57 – 1.52 (m, 7H), 1.13 – 1.04 (m, 13H), 1.00 – 0.93 (m, 5H); **^{13}C NMR (151 MHz, $CDCl_3$)** δ 162.07 (d, $^1J_{C\text{-}}$

$_F$ = 244.5 Hz), 145.61, 140.22, 139.77 (d, $^4J_{C\text{-}F}$ = 2.9 Hz), 130.14, 130.01, 129.18, 128.77, 128.37, 127.86 (d, $^3J_{C\text{-}F}$ = 7.9 Hz), 127.12, 117.07, 115.65 (d, $^2J_{C\text{-}F}$ = 21.2 Hz), 112.68, 57.82, 56.58, 54.05, 32.68, 31.03, 26.66, 26.63, 26.25; **IR (neat)**: v_{max} = 3299, 3032, 2926, 2851, 1603, 1505, 1488, 1220, 737, 701 cm^{-1}; **HRMS** m/z (FAB) calc. for $C_{32}H_{40}FN_2$ [M+H] = 471.3176, Found 471.3174; ***R**$_f$* 0.64 (hex/EtOAc, 9/1)

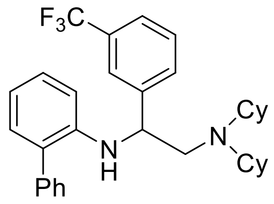

N[1]-([1,1'-Biphenyl]-2-yl)-*N*[2],*N*[2]-dicyclohexyl-1-(3-(trifluoromethyl)phenyl) ethane-1,2-diamine (**2m**): white solid (91%); mp 152-157 °C; **^{1}H NMR (600 MHz, $CDCl_3$)** δ 7.69 (s, 1H), 7.67 (d, *J* = 7.8 Hz, 1H), 7.57 – 7.55 (m, 3H), 7.51 – 7.47 (m, 3H), 7.37 (t, *J* = 7.4 Hz, 1H), 7.13 (dd, *J* = 7.4, 1.7 Hz, 1H), 7.04 (ddd, *J* = 8.2, 7.4, 1.7 Hz, 1H), 6.77 (ddd, *J* = 7.4, 7.4, 1.1 Hz, 1H), 6.24 (dd, *J* = 8.2, 1.1 Hz, 1H), 5.41 (s, 1H), 4.17 (dd, *J* = 10.7, 4.6 Hz, 1H), 2.92 (dd, *J* = 13.6, 4.6 Hz, 1H), 2.38 (dd, *J* = 13.6, 10.7 Hz, 1H), 2.35 – 2.30 (m, 2H), 1.70 – 1.66 (m, 4H), 1.59 – 1.54 (m, 4H), 1.15 – 1.08 (m, 8H), 1.02 – 0.96 (m, 4H); **^{13}C NMR (151 MHz, $CDCl_3$)** δ 145.52, 145.45, 140.12, 131.12 (q, $^2J_{C\text{-}F}$ = 32.0 Hz), 130.24, 130.03, 129.87, 129.41, 129.33, 128.82, 128.46, 127.21, 124.53 (q, $^1J_{C\text{-}F}$ = 272.3 Hz), 124.11 (q, $^3J_{C\text{-}F}$ = 3.8 Hz), 123.23 (q, $^3J_{C\text{-}F}$ = 3.8 Hz), 117.38, 112.66, 57.87, 57.24, 53.91, 32.63, 31.08, 26.64, 26.61, 26.22; **IR (neat)**: v_{max} = 3297, 3017, 2927, 2852, 1604, 1506, 1488, 1327, 1124, 738, 703 cm^{-1}; **HRMS** m/z (FAB) calc. for $C_{33}H_{40}F_3N_2$ [M+H] = 521.3144, Found 521.3142; ***R**$_f$* 0.63 (hex/EtOAc, 9/1)

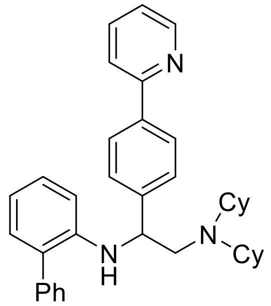

N[1]-([1,1'-Biphenyl]-2-yl)-*N*[2],*N*[2]-dicyclohexyl-1-(4-(pyridin-2-yl)phenyl)ethane-1,2-diamine (**2o**): white solid (52%); mp 175-180 °C; **^{1}H NMR (600 MHz, $CDCl_3$)** δ 8.71 (d, *J* = 4.8 Hz, 1H), 8.01 (d, *J* = 8.1 Hz, 2H), 7.76 – 7.73 (m, 2H), 7.58 (d, *J* = 7.8 Hz, 2H), 7.56 (d, *J* = 8.1 Hz, 2H), 7.47 (dd, *J* = 7.8, 7.4 Hz, 2H), 7.36 (t, *J* = 7.4 Hz, 1H), 7.22 (ddd, *J* = 5.3, 2.8 Hz, 1H), 7.11 (d, *J* = 7.2 Hz, 1H), 7.01 (dd, *J* = 8.1, 7.5 Hz, 1H), 6.73 (dd, *J* = 7.5, 7.2 Hz, 1H), 6.35 (d, *J* = 8.1 Hz, 1H), 5.43 (s, 1H), 4.17 (dd, *J* = 10.8, 4.4 Hz, 1H), 2.95 (dd, *J* = 13.6, 4.4 Hz, 1H), 2.42 (dd, *J* = 13.6, 10.8 Hz, 1H), 2.37 – 2.32 (m, 2H), 1.70 – 1.65 (m, 4H), 1.61 – 1.53 (m, 4H), 1.15 – 1.08 (m, 8H), 1.02 – 0.95 (m, 4H); **^{13}C NMR (151 MHz, $CDCl_3$)** δ 157.68, 149.83, 145.70, 145.31, 140.29, 138.41, 136.83, 130.07, 130.03, 129.07, 128.74, 128.40, 127.48, 127.07, 126.87, 122.06, 120.57, 116.97, 112.89, 57.79, 57.08, 53.84, 32.69, 30.98, 26.66, 26.62, 26.25; **IR (neat)**: v_{max} = 3306, 2987, 2972, 2927, 2854, 1586, 1505, 1488, 1435, 750, 701

cm^{-1}; **HRMS** m/z (FAB) calc. for $C_{37}H_{44}N_3$ [M+H] = 530.3535, Found 530.3532; $\boldsymbol{R_f}$ 0.25 (hex/EtOAc, 9/1)

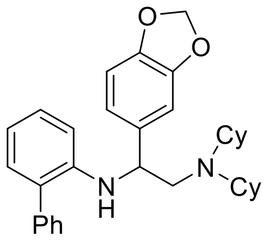

N[1]-([1,1'-Biphenyl]-2-yl)-1-(benzo[*d*][1,3]dioxol-5-yl)-*N*[2],*N*[2]-dicyclohexylethane-1,2-diamine (**2p**): pale yellow solid (36%); mp 80-85 °C; **^{1}H NMR (600 MHz, CDCl$_3$)** δ 7.55 (d, *J* = 7.6 Hz, 2H), 7.46 (dd, *J* = 7.6, 7.4 Hz, 2H), 7.36 (t, *J* = 7.4 Hz, 1H), 7.11 (dd, *J* = 7.3, 1.7 Hz, 1H), 7.06 (ddd, *J* = 8.2, 7.2, 1.7 Hz, 1H), 6.98 (s, 1H), 6.93 (d, *J* = 7.8 Hz, 1H), 6.83 (d, *J* = 7.8 Hz, 1H), 6.75 (dd, *J* = 7.3, 7.2 Hz, 1H), 6.39 (d, *J* = 8.2 Hz, 1H), 5.97 (d, *J* = 1.6 Hz, 1H), 5.95 (d, *J* = 1.6 Hz, 1H), 5.36 (s, 1H), 4.04 (dd, *J* = 10.7, 4.4 Hz, 1H), 2.87 (dd, *J* = 13.6, 4.4 Hz, 1H), 2.36 (dd, *J* = 13.6, 10.7 Hz, 1H), 2.33 – 2.26 (m, 2H), 1.70 – 1.63 (m, 4H), 1.60 – 1.53 (m, 4H), 1.15 – 1.04 (m, 8H), 1.02 – 0.94 (m, 4H); **^{13}C NMR (151 MHz, CDCl$_3$)** δ 153.53, 150.31, 145.06, 139.97, 131.06, 130.27, 129.94, 129.26, 128.99, 128.80, 128.39, 127.21, 121.73, 117.47, 112.50, 57.83, 56.56, 53.22, 32.59, 31.02, 26.58, 26.55, 26.16; **IR (neat)**: v_{max} = 3300, 3013, 2925, 2851, 1603, 1504, 1485, 1242, 1039, 737, 701 cm^{-1}; **HRMS** m/z (FAB) calc. for $C_{33}H_{41}N_2O_2$ [M+H] = 497.3168; $\boldsymbol{R_f}$ 0.55 (hex/EtOAc, 9/1)

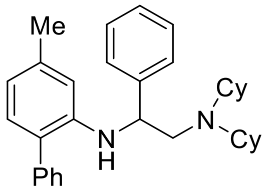

N[2],*N*[2]-Dicyclohexyl-*N*[1]-(4-methyl-[1,1'-biphenyl]-2-yl)-1-phenylethane-1,2 -diamine (**2q**): white solid (78%); mp 144-146 °C; **^{1}H NMR (600 MHz, CDCl$_3$)** δ 7.51 (d, *J* = 7.5 Hz, 2H), 7.44 – 7.40 (m, 4H), 7.35 (dd, *J* = 7.5, 7.5 Hz, 2H), 7.31 (t, *J* = 7.5 Hz, 1H), 7.25 (t, *J* = 7.4 Hz, 1H), 6.97 (d, *J* = 7.5 Hz, 1H), 6.52 (d, *J* = 7.5 Hz, 1H), 6.10 (s, 1H), 5.31 (s, 1H), 4.08 (dd, *J* = 10.7, 4.4 Hz, 1H), 2.87 (dd, *J* = 13.6, 4.4 Hz, 1H), 2.35 (dd, *J* = 13.6, 10.7 Hz, 1H), 2.32 – 2.27 (m, 2H), 2.12 (s, 3H), 1.63 (t, *J* = 13.1 Hz, 4H), 1.55 – 1.50 (m, 4H), 1.11 – 1.03 (m, 8H), 0.98 – 0.91 (m, 4H); **^{13}C NMR (151 MHz, CDCl$_3$)** δ 145.67, 144.37, 140.36, 138.06, 130.17, 129.96, 128.81, 128.72, 127.01, 126.90, 126.47, 126.45, 117.64, 113.53, 57.75, 57.09, 54.02, 32.70, 31.01, 26.69, 26.65, 26.29, 21.84; **IR (neat)**: v_{max} = 3306, 3024, 2927, 2852, 1615, 1516, 1489, 766, 700 cm^{-1}; $\boldsymbol{R_f}$ 0.56 (hex/EtOAc, 9/1)

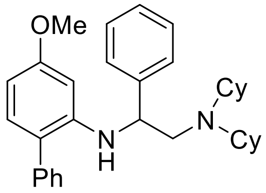

N^2,N^2-Dicyclohexyl-N^1-(4-methoxy-[1,1'-biphenyl]-2-yl)-1-phenylethane-1,2-diamine (**2r**): white solid (85%); mp 153-158 °C; **^{1}H NMR (600 MHz, $CDCl_3$)** δ 7.50 (dd, *J* = 8.0, 1.2 Hz, 2H), 7.44 – 7.40 (m, 4H), 7.35 (dd, *J* = 8.0, 7.2 Hz, 2H), 7.30 (t, *J* = 7.7 Hz, 1H), 7.24 (t, *J* = 7.2 Hz, 1H), 6.99 (d, *J* = 8.2 Hz, 1H), 6.26 (dd, *J* = 8.2, 2.4 Hz, 1H), 5.88 (d, *J* = 2.4 Hz, 1H), 5.41 (s, 1H), 4.08 (dd, *J* = 10.9, 4.4 Hz, 1H), 3.57 (s, 3H), 2.88 (dd, *J* = 13.6, 4.4 Hz, 1H), 2.37 (dd, *J* = 13.6, 10.9 Hz, 1H), 2.32 – 2.28 (m, 2H), 1.66 – 1.61 (m, 4H), 1.56 – 1.51 (m, 4H), 1.11 – 1.05 (m, 8H), 0.99 – 0.92 (m, 4H); **^{13}C NMR (151 MHz, $CDCl_3$)** δ 160.05, 147.06, 144.09, 140.10, 130.62, 130.26, 128.89, 128.72, 127.16, 126.80, 126.49, 122.30, 101.35, 99.52, 57.83, 57.33, 55.01, 53.92, 32.69, 31.03, 26.68, 26.65, 26.28; **IR (neat)**: ν_{max} = 3303, 3024, 2927, 2851, 1613, 1515, 1488, 1305, 1212, 1171, 765, 701 cm^{-1}; ***R_f*** 0.46 (hex/EtOAc, 9/1)

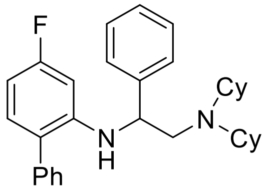

N^2,N^2-Dicyclohexyl-N^1-(4-fluoro-[1,1'-biphenyl]-2-yl)-1-phenylethane-1,2-diamine (**2s**): pale yellow solid (83%); mp 140-147 °C; **^{1}H NMR (600 MHz, $CDCl_3$)** δ 7.47 (d, *J* = 7.3 Hz, 2H), 7.43 (dd, *J* = 7.5, 7.3 Hz, 2H), 7.40 (d, *J* = 7.3 Hz, 2H), 7.36 (dd, *J* = 7.3, 7.2 Hz, 2H), 7.33 (t, *J* = 7.5 Hz, 1H), 7.28 (t, *J* = 7.2 Hz, 1H), 6.98 (dd, *J* = 8.3 Hz, $^4J_{H\text{-}F}$ = 6.7 Hz, 1H), 6.36 (ddd, $^3J_{H\text{-}F}$ = 8.3 Hz, *J* = 8.3, 2.5 Hz, 1H), 5.98 (dd, $^3J_{H\text{-}F}$ =12.1 Hz, *J* = 2.5 Hz, 1H), 5.46 (s, 1H), 4.03 (dd, *J* = 10.8, 4.5 Hz, 1H), 2.88 (dd, *J* = 13.6, 4.5 Hz, 1H), 2.34 (dd, *J* = 13.6, 10.8 Hz, 1H), 2.30 – 2.25 (m, 2H), 1.66 – 1.61 (m, 4H), 1.54 – 1.51 (m, 4H), 1.11 – 1.02 (m, 8H), 0.97 – 0.91 (m, 4H); **^{13}C NMR (151 MHz, $CDCl_3$)** δ 163.47 (d, $^1J_{C\text{-}F}$ = 242.2 Hz), 147.57 (d, $^3J_{C\text{-}F}$ = 11.0 Hz), 143.42, 139.52, 130.80 (d, $^3J_{C\text{-}F}$ = 9.8 Hz), 130.16, 128.99, 128.87, 127.36, 127.23, 126.40, 124.97, 102.94 (d, $^2J_{C\text{-}F}$ = 21.5 Hz), 99.92 (d, $^2J_{C\text{-}F}$ = 26.1 Hz), 57.91, 57.23, 53.85, 32.66, 31.08, 26.66, 26.62, 26.25; **IR (neat)**: ν_{max} = 3287, 2987, 2901, 1615, 1513, 1488, 1066, 751, 700 cm^{-1}; ***R_f*** 0.58 (hex/EtOAc, 9/1)

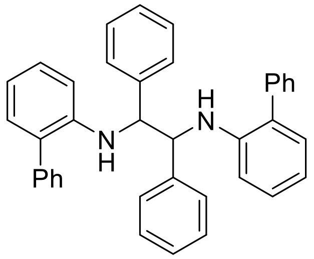

N^1,N^2-di([1,1'-Biphenyl]-2-yl)-1,2-diphenylethane-1,2-diamine (**3a**): white solid (75%); mp 154-162 °C; **^{1}H NMR (600 MHz, $CDCl_3$)** δ 7.50 (dd, *J* = 7.5 Hz, 2H), 7.45 – 7.36 (m, 4H), 7.28 (d, *J* = 7.5 Hz, 2H), 7.25 (d, *J* = 8.4 Hz, 2H), 7.20 – 7.16 (m, 3H), 7.13 (t, *J* = 7.3 Hz, 1H), 7.07 – 6.99 (m, 7H), 6.93 (t, *J* = 7.8 Hz, 1H), 6.70 (t, *J* = 7.4 Hz, 1H), 6.68 – 6.65 (m, 3H), 6.34 (d, *J* = 8.2 Hz, 1H), 6.09 (d, *J* = 8.2 Hz, 1H), 4.81 (d, *J* = 6.9 Hz, 1H), 4.68 (d, *J* = 6.9 Hz, 1H), 4.61 (s, 1H), 4.49 (s, 1H); **^{13}C NMR (151 MHz, $CDCl_3$)**

δ 143.96, 143.47, 139.83, 139.44, 139.16, 138.40, 130.05, 129.81, 129.51, 129.13, 128.97, 128.87, 128.63, 128.62, 128.48, 128.35, 128.33, 127.64, 127.58, 127.51, 127.39, 126.75, 117.45, 117.39, 111.90, 111.75, 64.11, 62.47; **IR (neat)**: ν_{max} = 3412, 3060, 3027, 2923, 1602, 1507, 1489, 1437, 744, 702 cm^{-1}; ***R$_f$*** 0.41 (hex/EtOAc, 9/1)

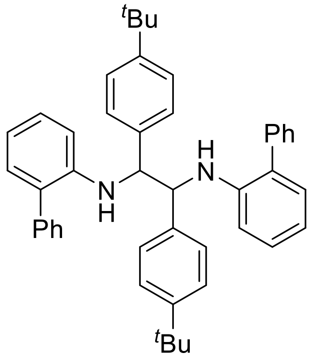

N[1],*N*[2]-di([1,1'-Biphenyl]-2-yl)-1,2-di(naphthalen-1-yl)ethane-1,2-diamine (**3b**): white solid (85%); mp 160-167 °C; **^{1}H NMR (600 MHz, CDCl$_3$)** δ 7.49 (t, *J* = 7.5 Hz, 2H), 7.44 – 7.39 (m, 4H), 7.25 – 7.21 (m, 4H), 7.14 (d, *J* = 8.0 Hz, 2H), 7.06 (d, *J* = 8.0 Hz, 2H), 7.01 – 6.93 (m, 6H), 6.68 – 6.64 (m, 4H), 6.35 (d, *J* = 8.2 Hz, 1H), 6.09 (d, *J* = 8.2 Hz, 1H), 4.61 – 4.59 (m, 2H), 4.54 (s, 1H), 4.43 (s, 1H), 1.29 (s, 9H), 1.27 (s, 9H); **^{13}C NMR (151 MHz, CDCl$_3$)** δ 150.24, 144.30, 143.82, 139.51, 139.24, 136.60, 135.84, 129.98, 129.88, 129.68, 129.59, 129.05, 128.90, 128.60, 128.57, 128.47, 128.33, 127.52, 127.41, 126.98, 126.22, 125.80, 125.27, 117.14, 117.12, 111.93, 111.72, 63.68, 62.49, 34.61, 34.58, 31.55, 31.54; **IR (neat)**: ν_{max} = 3407, 3057, 2962, 2904, 1605, 1507, 1489, 746, 703 cm^{-1}; ***R$_f$*** 0.51 (hex/EtOAc, 9/1)

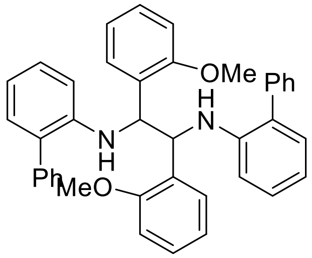

N[1],*N*[2]-di([1,1'-Biphenyl]-2-yl)-1,2-bis(2-methoxyphenyl)ethane-1,2-diamine (**3c**): white solid (81%); mp 158-165 °C; **^{1}H NMR (600 MHz, CDCl$_3$)** δ 7.47 (dd, *J* = 7.5 Hz, 2H), 7.42 – 7.33 (m, 5H), 7.27 (d, *J* = 7.0 Hz, 2H), 7.24 – 7.22 (m, 2H), 7.09 – 6.89 (m, 7H), 6.73 – 6.64 (m, 5H), 6.61 (d, *J* = 7.6 Hz, 1H), 6.36 (d, *J* = 8.2 Hz, 1H), 6.34 (d, *J* = 7.6 Hz, 1H), 6.20 (t, *J* = 2.1 Hz, 1H), 6.16 (d, *J* = 8.3 Hz, 1H), 4.75 (s, 1H), 4.47 (s, 1H), 3.67 (s, 3H), 3.56 (s, 3H); **^{13}C NMR (151 MHz, CDCl$_3$)** δ 160.05, 159.51, 143.42, 140.26, 139.40, 139.20, 130.10, 129.91, 129.87, 129.70, 129.44, 129.43, 129.11, 128.97, 128.65, 128.52, 128.38, 127.55, 127.51, 119.51, 119.12, 117.52, 113.41, 113.08, 113.00, 112.66, 111.89, 64.05, 62.56, 55.27, 55.14; **IR (neat)**: ν_{max} = 3396, 2987, 2901, 1600, 1505, 1488, 1257, 1050, 749, 704 cm^{-1}; ***R$_f$*** 0.21 (hex/EtOAc, 9/1)

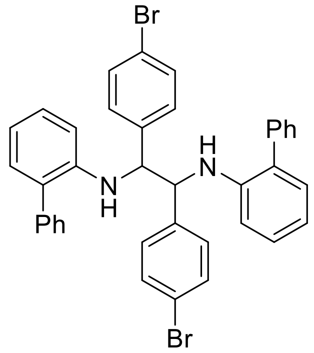

N[1],*N*[2]-di([1,1'-Biphenyl]-2-yl)-1,2-bis(4-bromophenyl)ethane-1,2-diamine (**3d**): pale yellow solid (78%); mp 161-165 °C; **^{1}H NMR (600 MHz, $CDCl_3$)** δ 7.49 (dd, J = 7.4 Hz, 2H), 7.43 (t, J = 7.4 Hz, 1H), 7.40 – 7.37 (m, 3H), 7.27 (d, J = 7.2 Hz, 2H), 7.23 (dd, J = 8.2, 1.5 Hz, 2H), 7.20 – 7.18 (m, 4H), 7.05 – 6.99 (m, 3H), 6.94 (ddd, J = 8.3, 1.6 Hz, 1H), 6.86 (d, J = 8.4 Hz, 2H), 6.73 – 6.68 (m, 2H), 6.52 (d, J = 8.4 hHz, 2H), 6.25 (d, J = 8.0 Hz, 1H), 6.06 (d, J = 8.0 Hz, 1H), 4.70 (s, 1H), 4.61 – 4.40 (m, 2H), 4.39 (s, 1H); **^{13}C NMR (151 MHz, $CDCl_3$)** δ 143.42, 142.97, 139.18, 138.95, 138.59, 137.32, 132.04, 131.66, 130.16, 129.97, 129.72, 129.44, 129.21, 129.07, 129.03, 128.78, 128.69, 128.59, 128.54, 128.48, 127.78, 127.69, 121.66, 121.64, 118.07, 117.95, 111.88, 111.76, 63.44, 62.02; **IR (neat)**: v_{max} = 2988, 2901, 1653, 1505, 1485, 764, 748, 419 cm^{-1}; ***R_f*** 0.43 (hex/EtOAc, 9/1)

NMR spectra

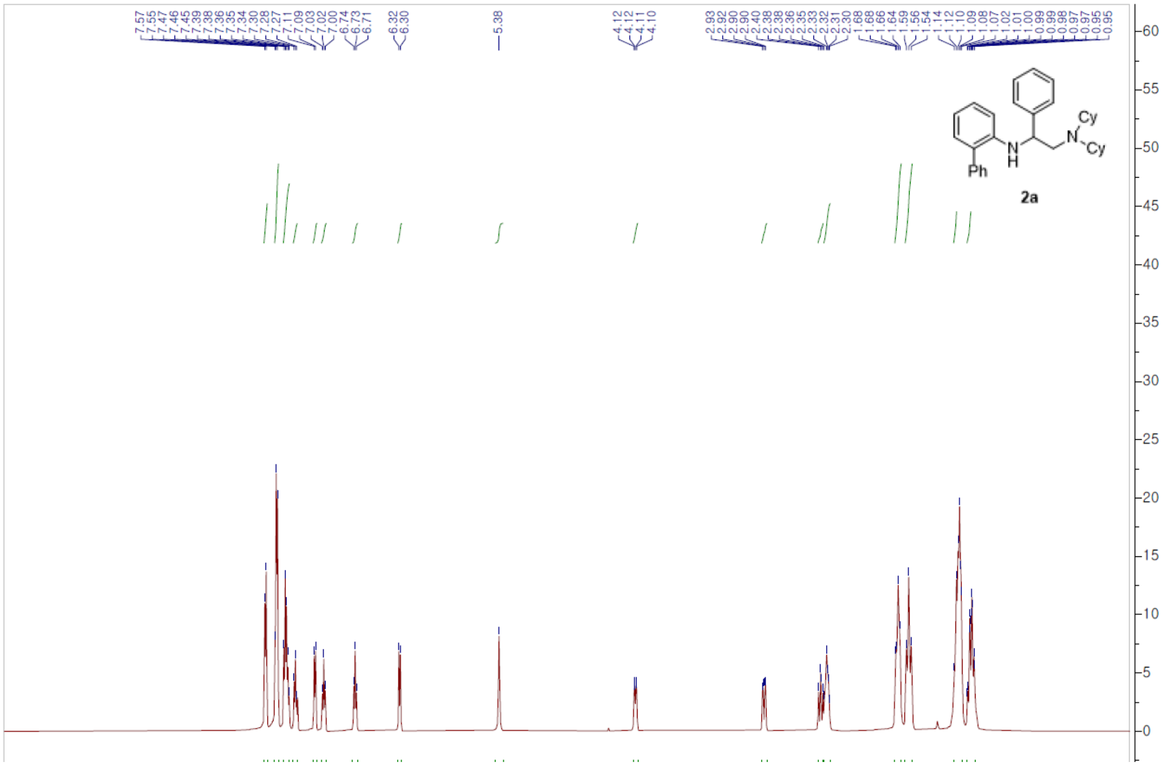

2a

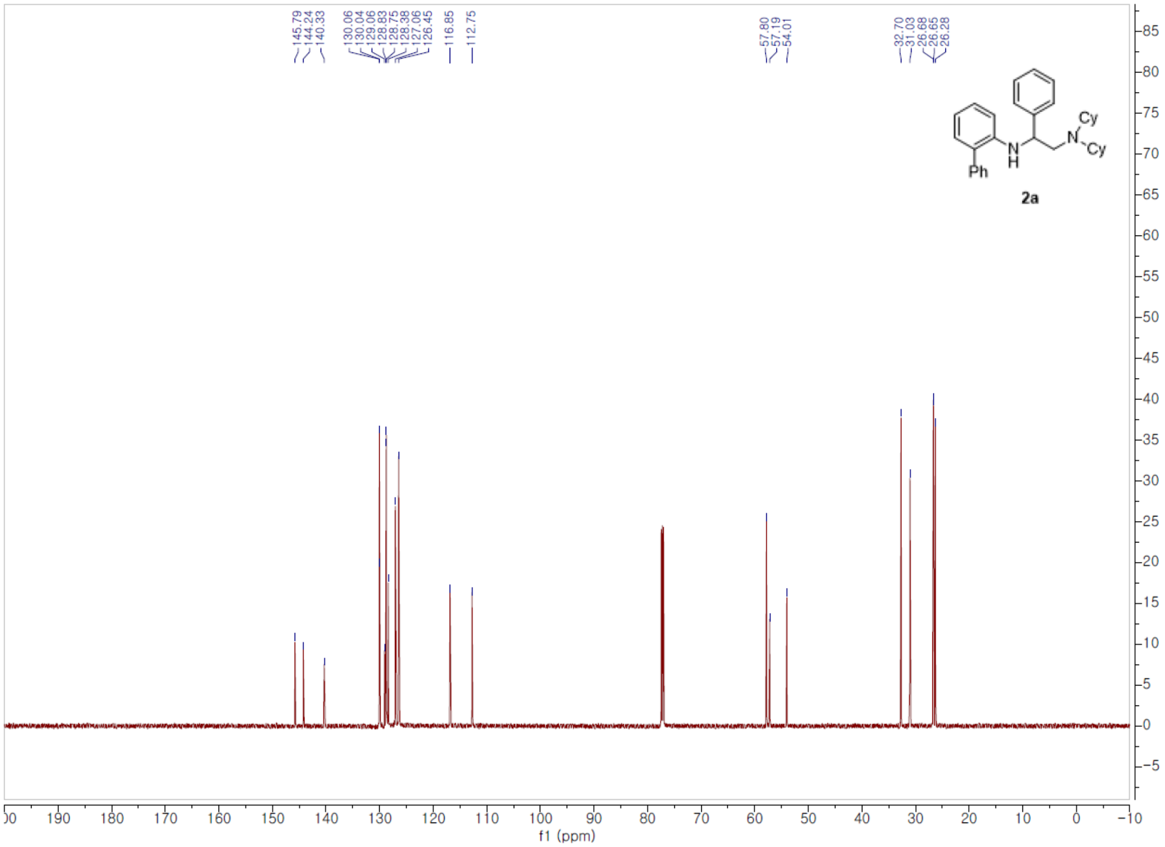

2a

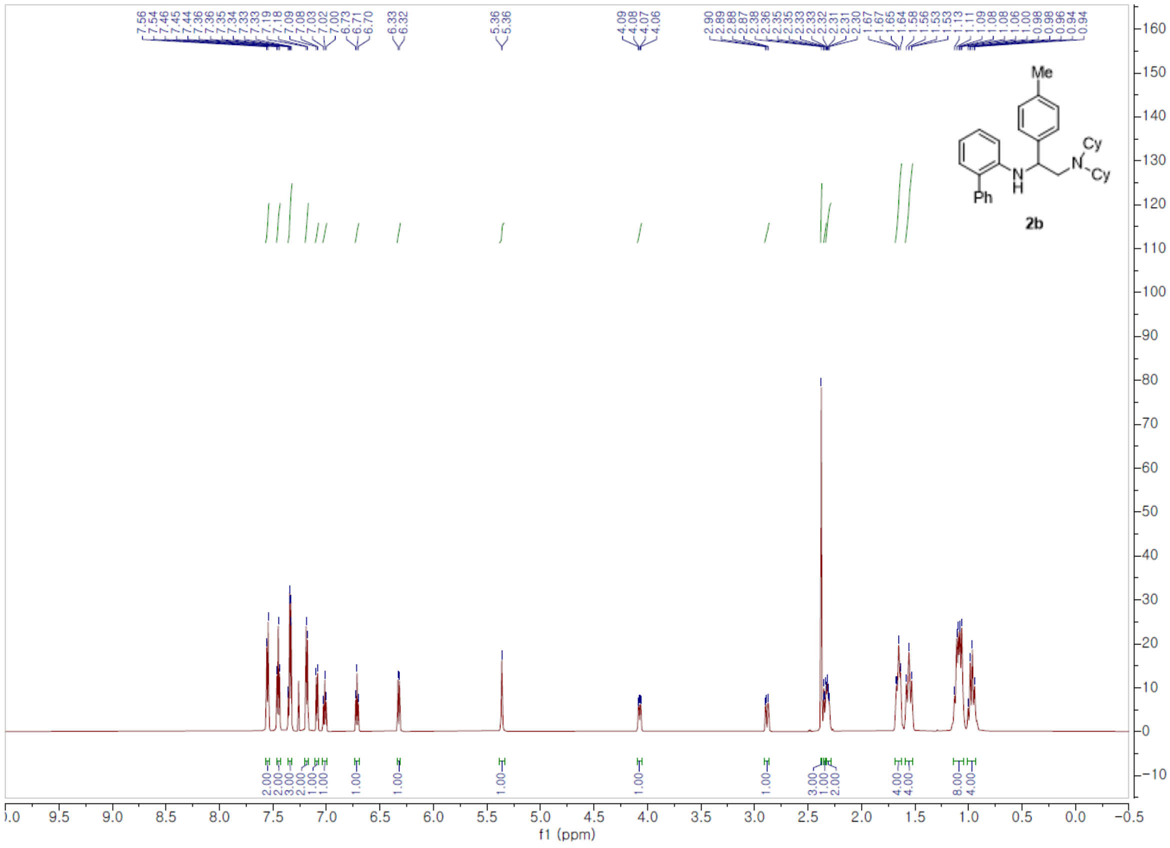

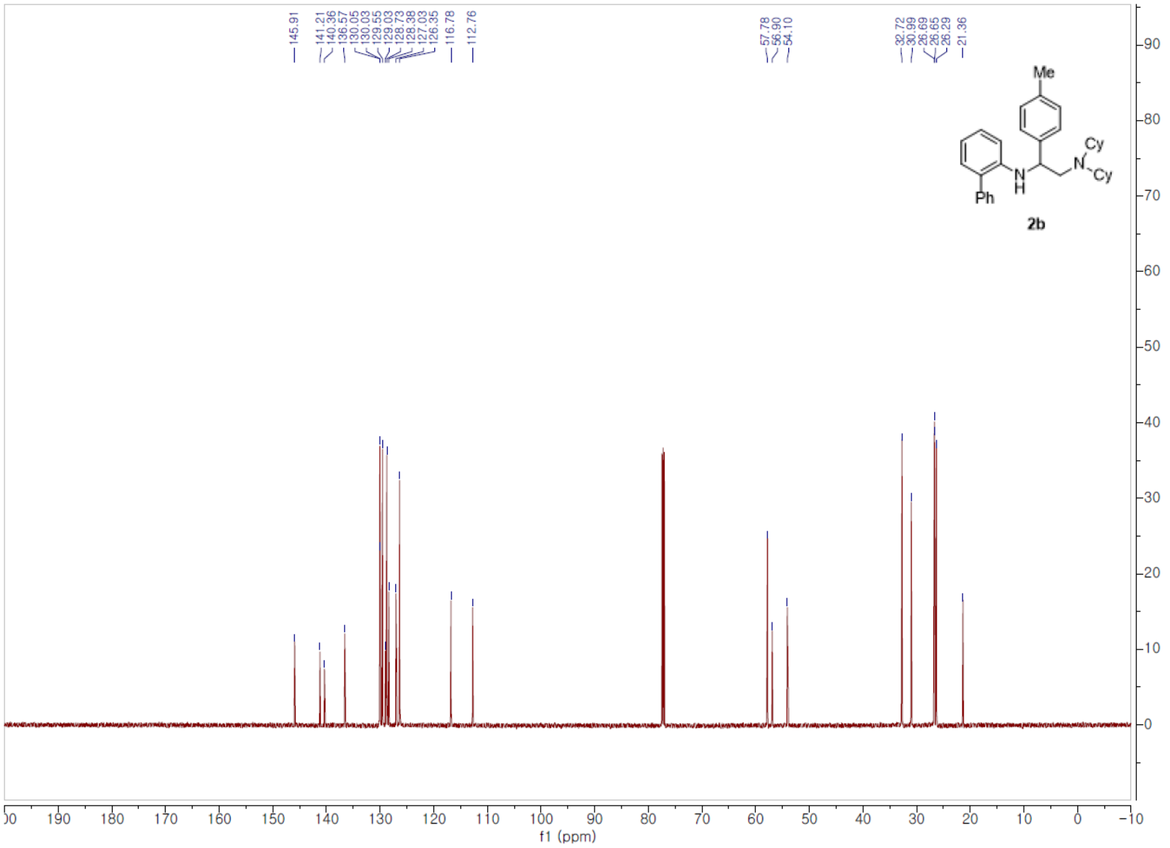

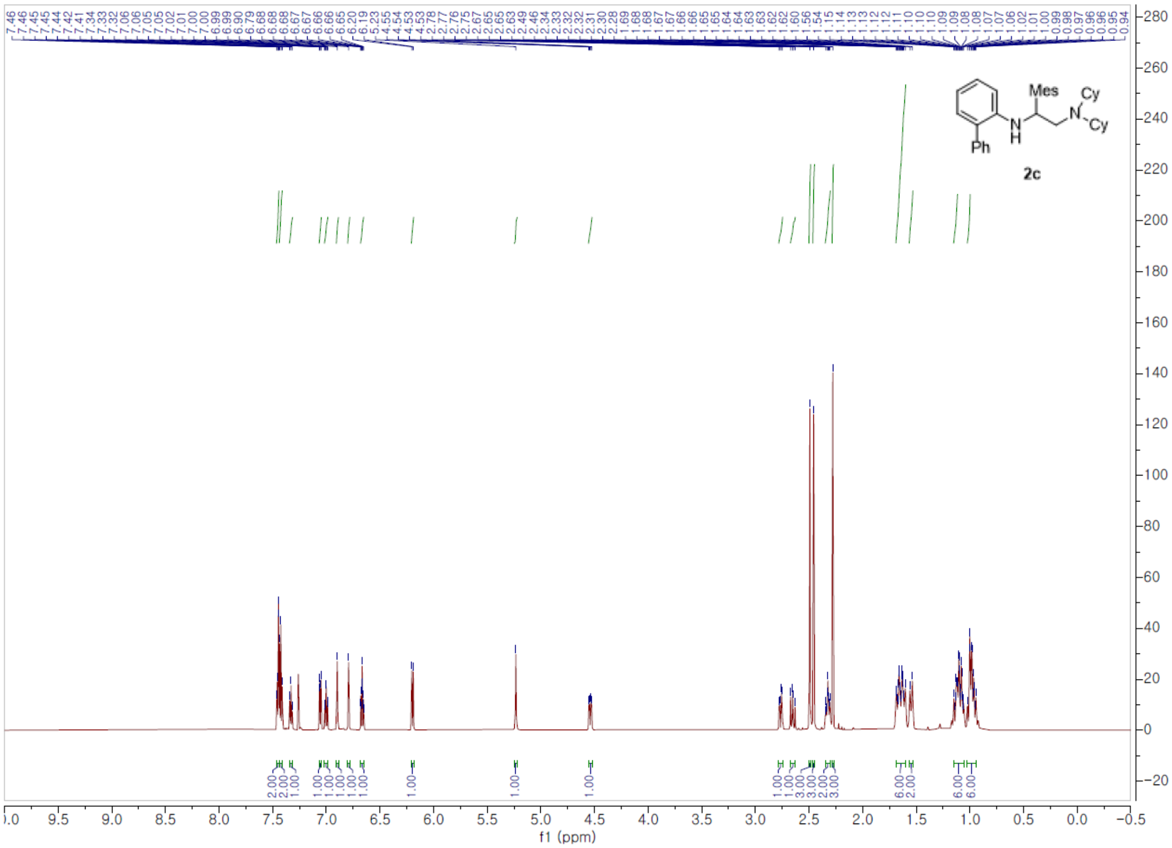

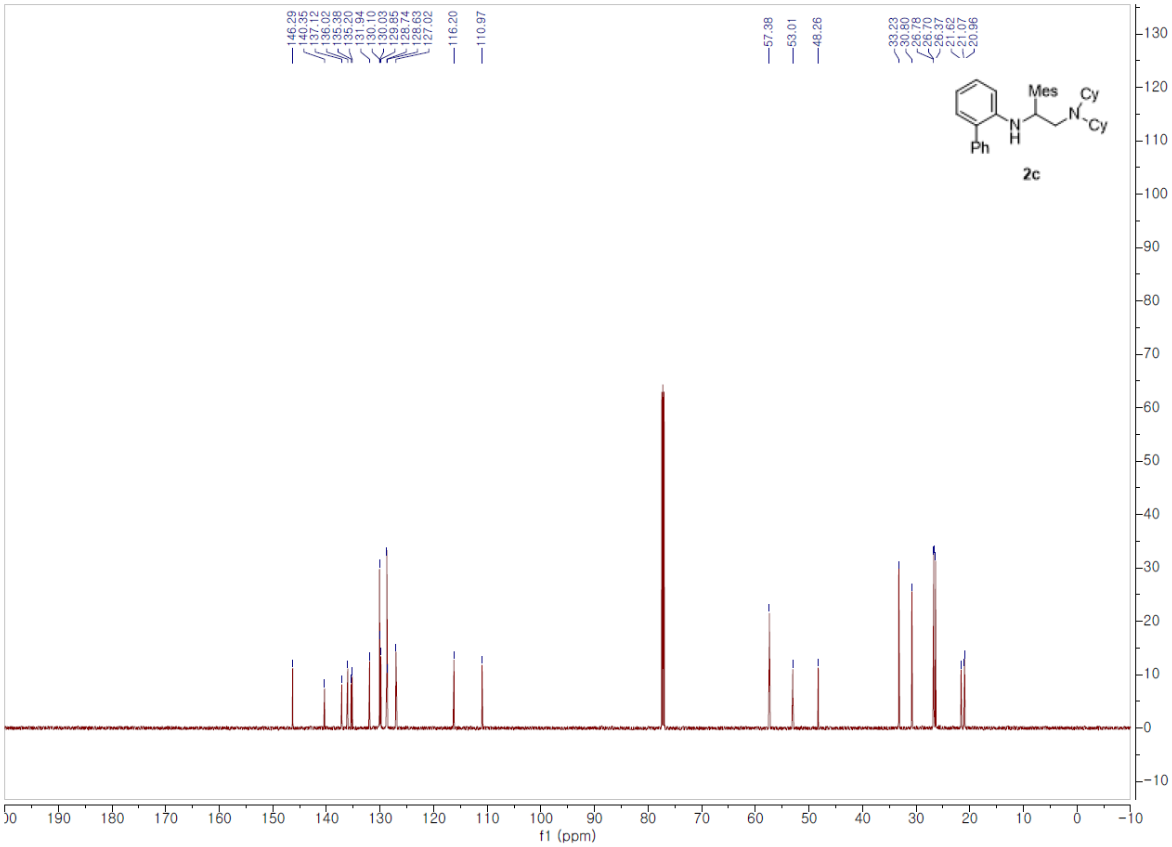

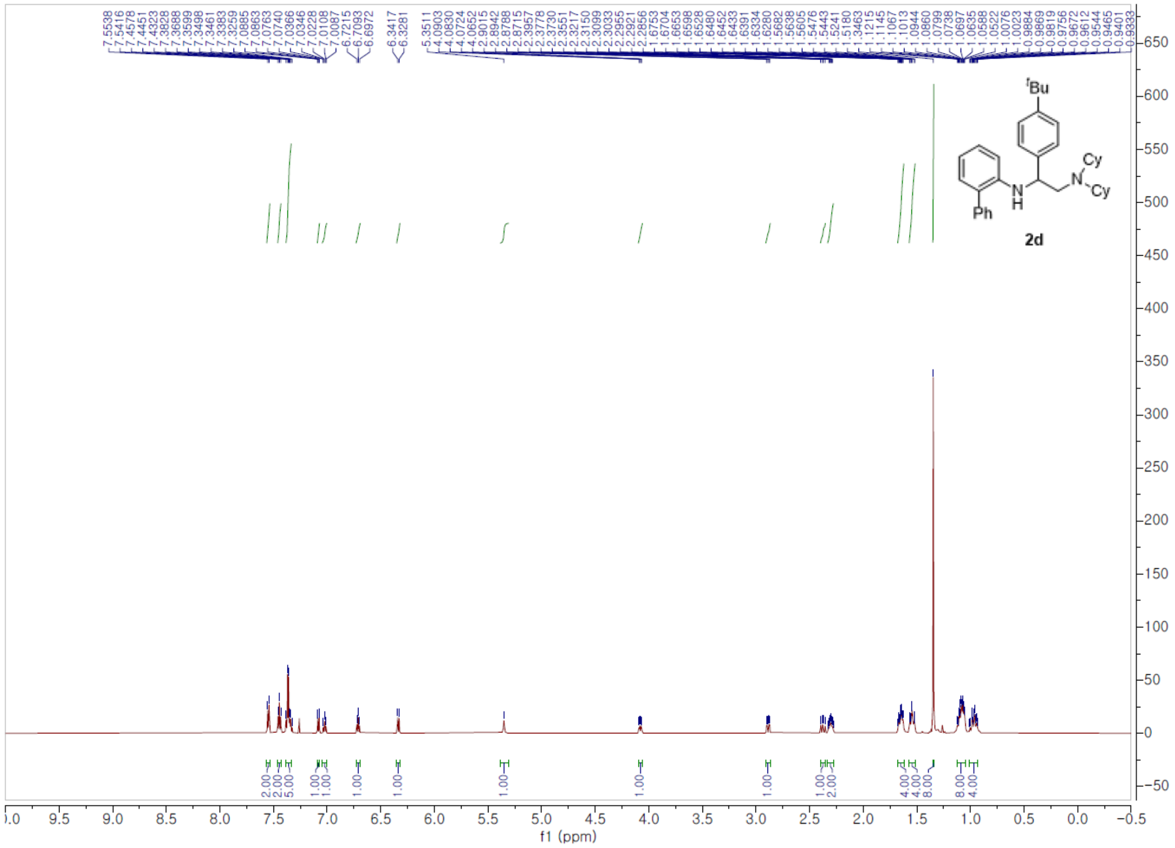

ᵗBu
N
H
Cy
N
Cy
Ph
2d
f1 (ppm)

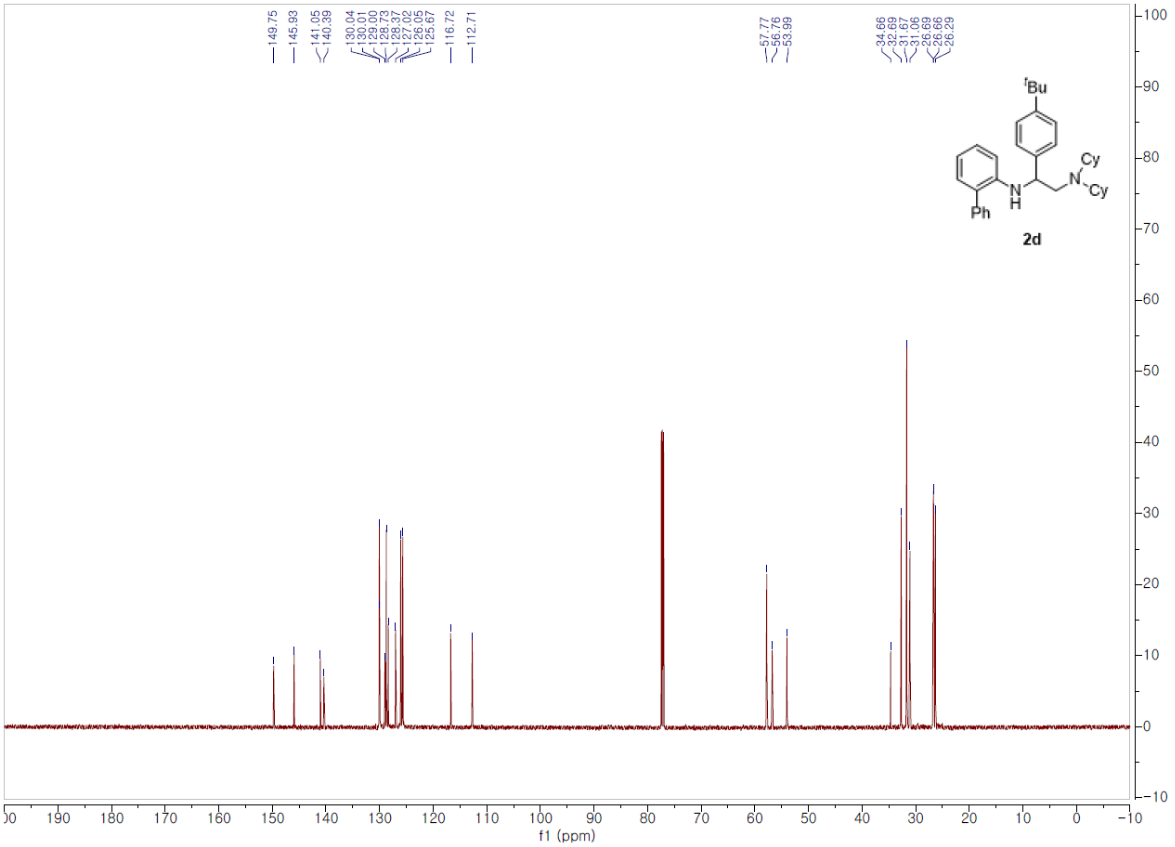

ᵗBu
N
H
Cy
N
Cy
Ph
2d
f1 (ppm)

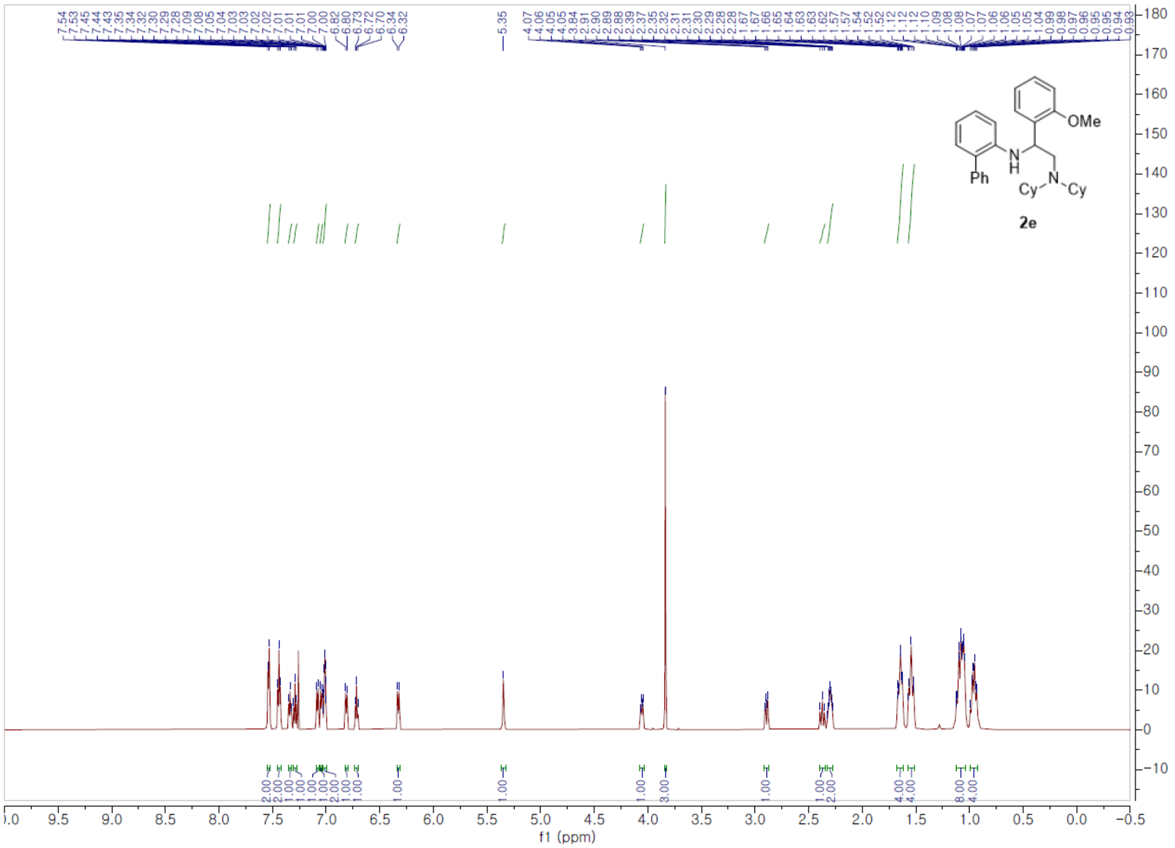
7.54
7.53
7.45
7.44
7.43
7.35
7.34
7.32
7.30
7.29
7.28
7.09
7.08
7.05
7.04
7.03
7.03
7.02
7.02
7.01
7.01
7.01
7.00
7.00
6.82
6.80
6.73
6.72
6.70
6.34
6.32
5.35
4.07
4.06
4.05
4.05
3.84
2.91
2.90
2.89
2.88
2.39
2.37
2.35
2.32
2.31
2.31
2.30
2.29
2.28
2.28
1.67
1.67
1.66
1.65
1.64
1.63
1.63
1.62
1.57
1.57
1.54
1.52
1.52
1.12
1.12
1.12
1.10
1.09
1.08
1.08
1.07
1.07
1.06
1.06
1.05
1.05
1.04
0.99
0.98
0.97
0.96
0.95
0.95
0.94
0.93
OMe
N
H
Ph
Cy
N
Cy
2e
f1 (ppm)

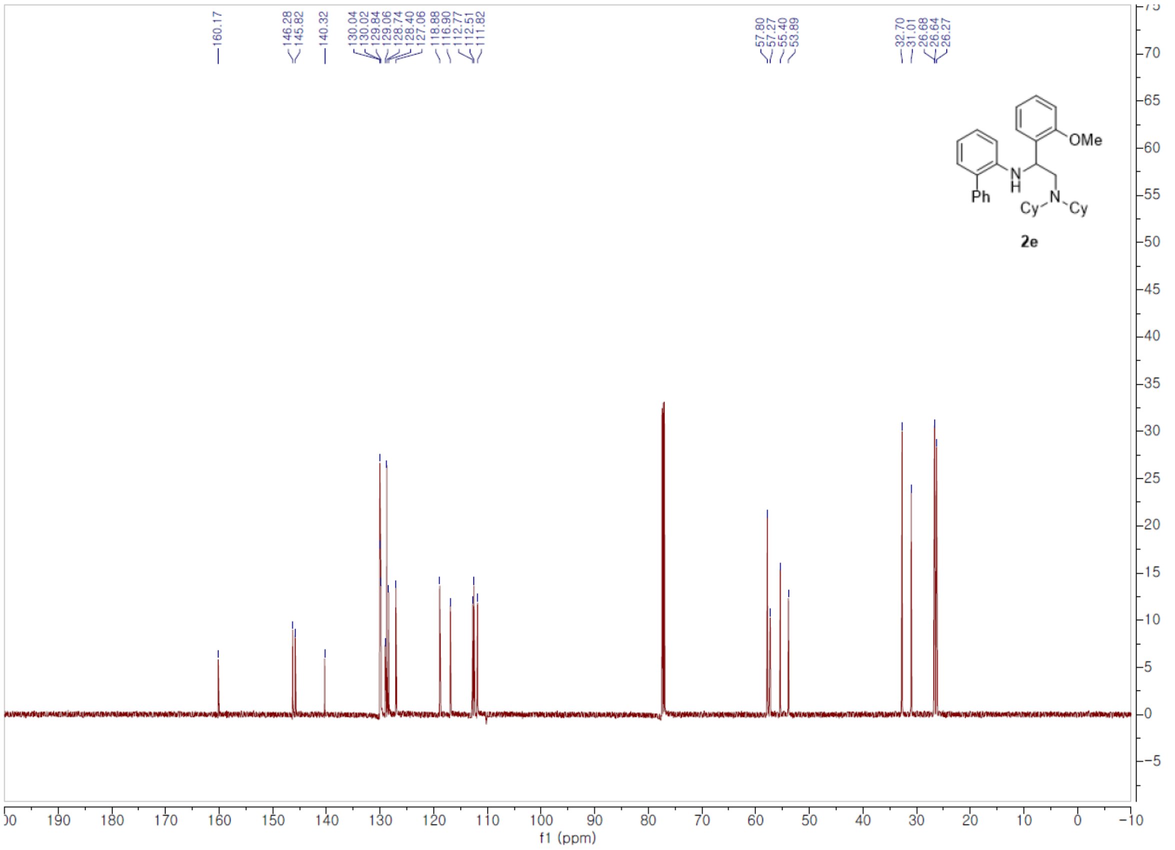
160.17
146.28
145.82
140.32
130.04
130.02
129.64
129.06
128.74
128.40
127.06
118.88
116.90
112.77
112.51
111.82
57.80
57.27
55.40
53.89
32.70
31.01
26.68
26.64
26.27
OMe
N
H
Ph
Cy
N
Cy
2e
f1 (ppm)

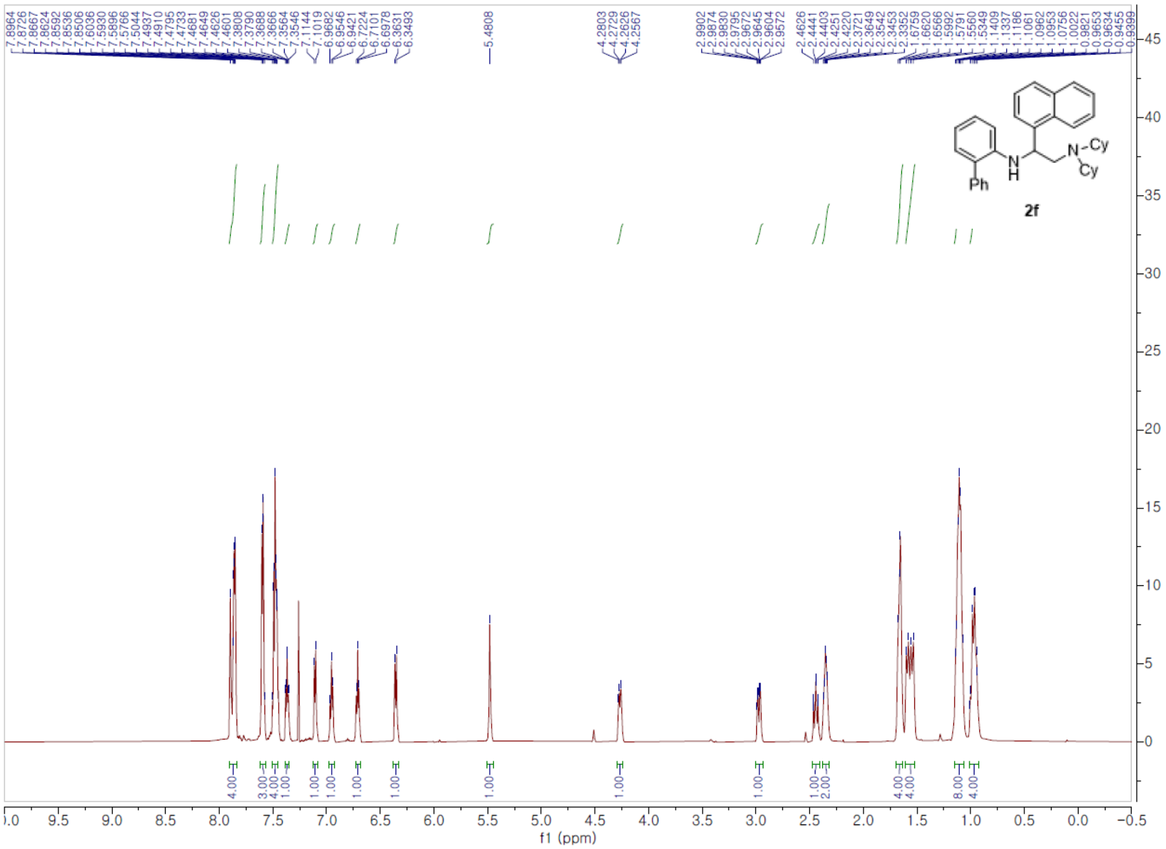

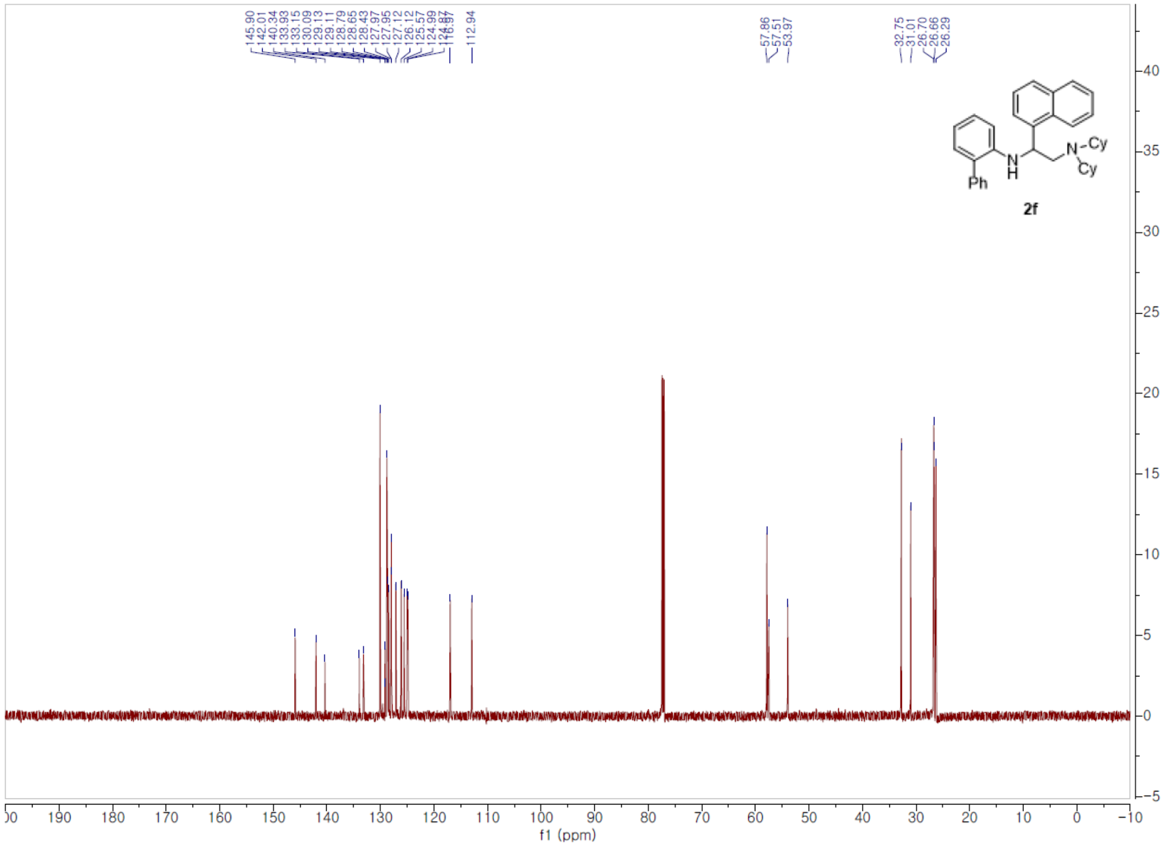

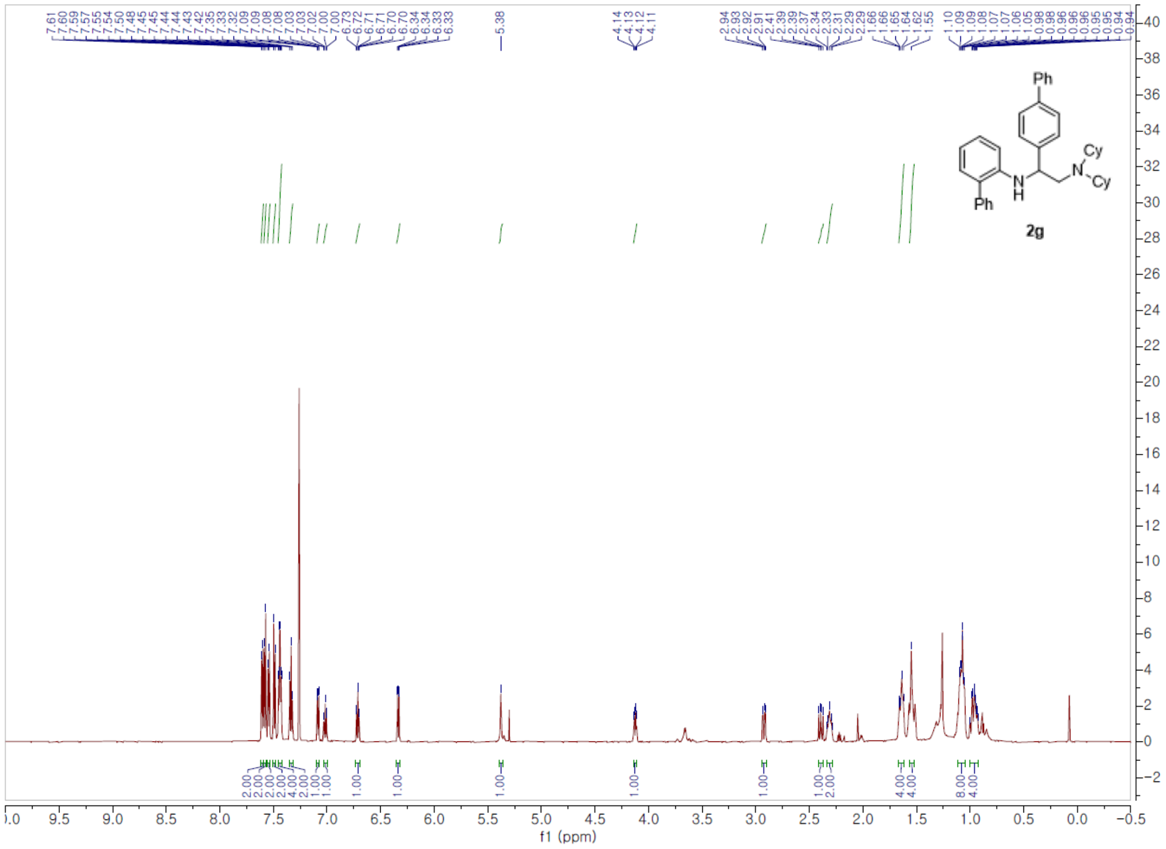

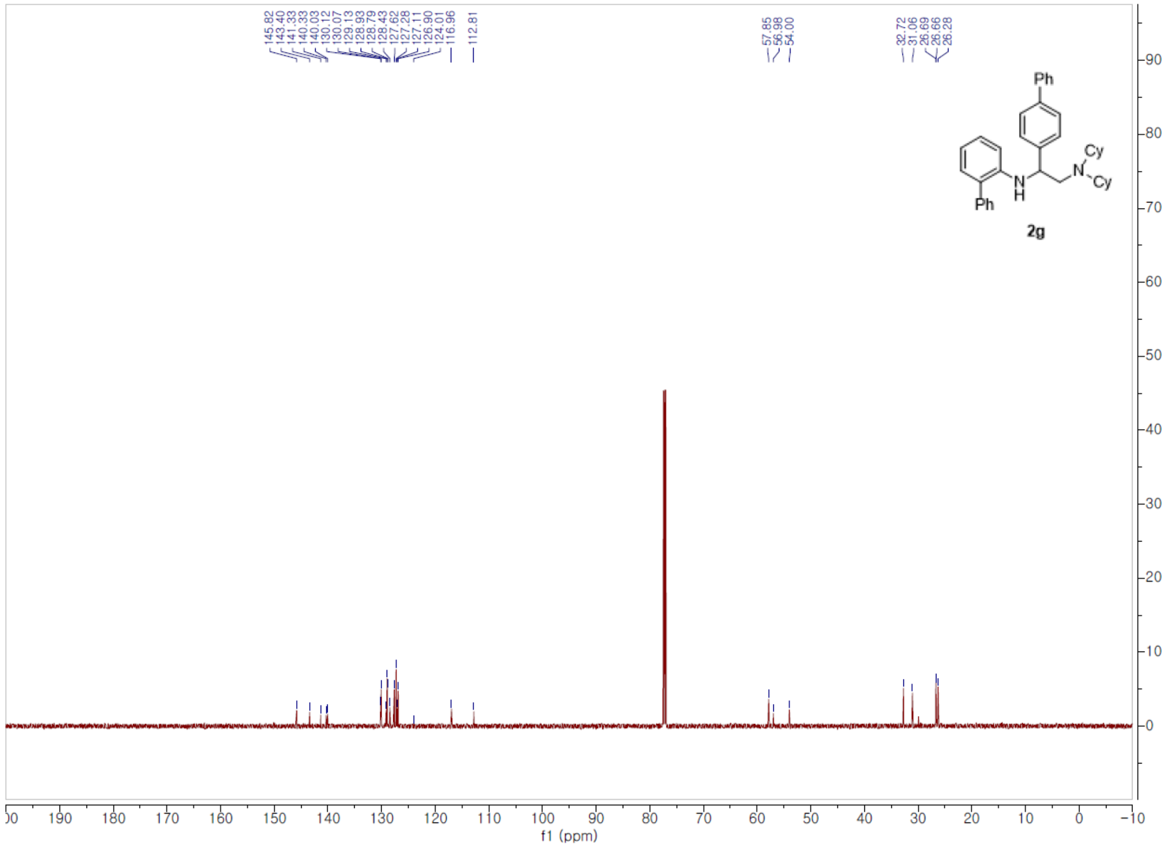

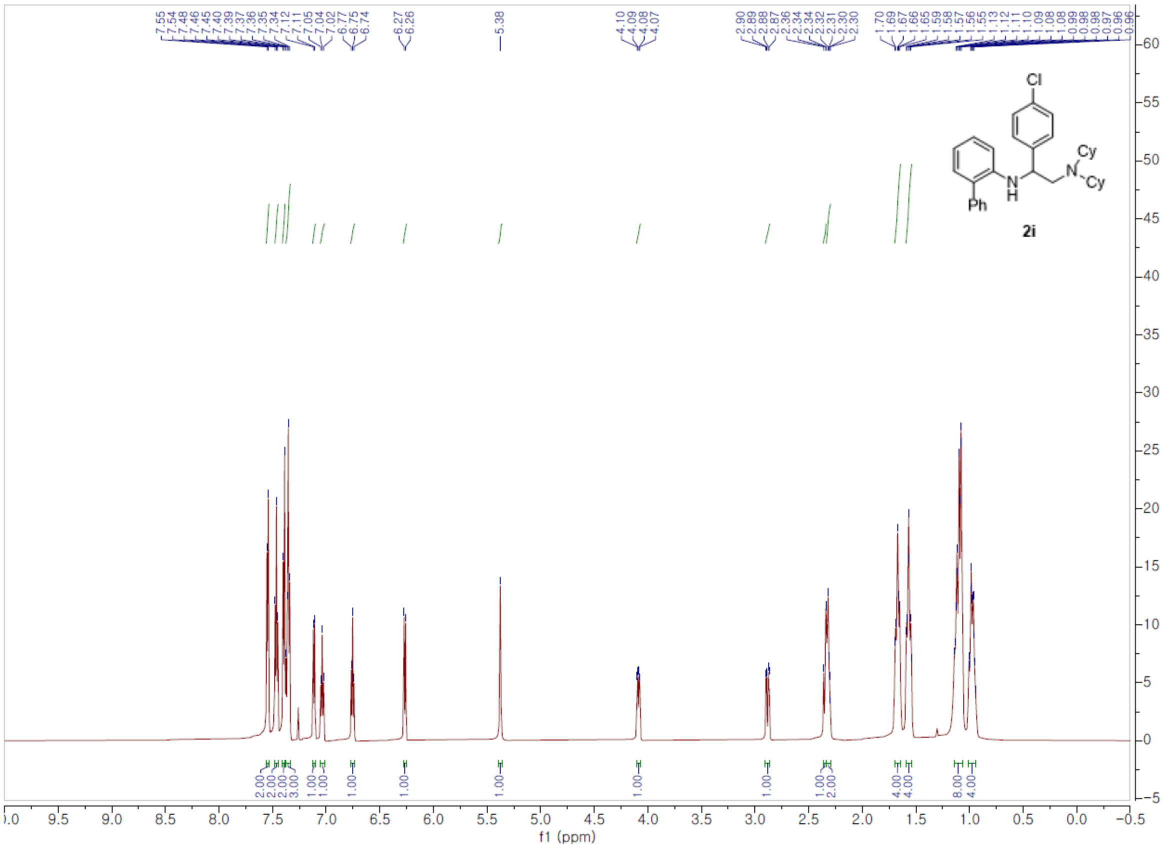

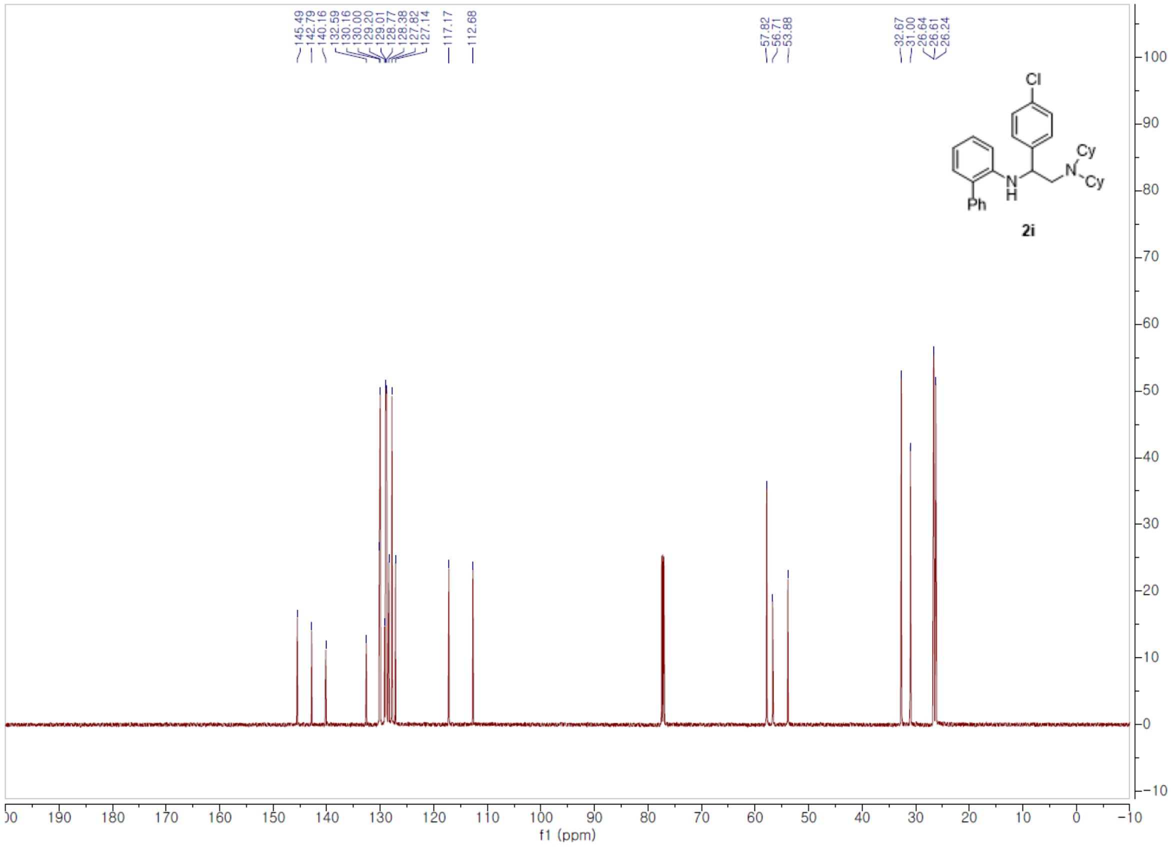

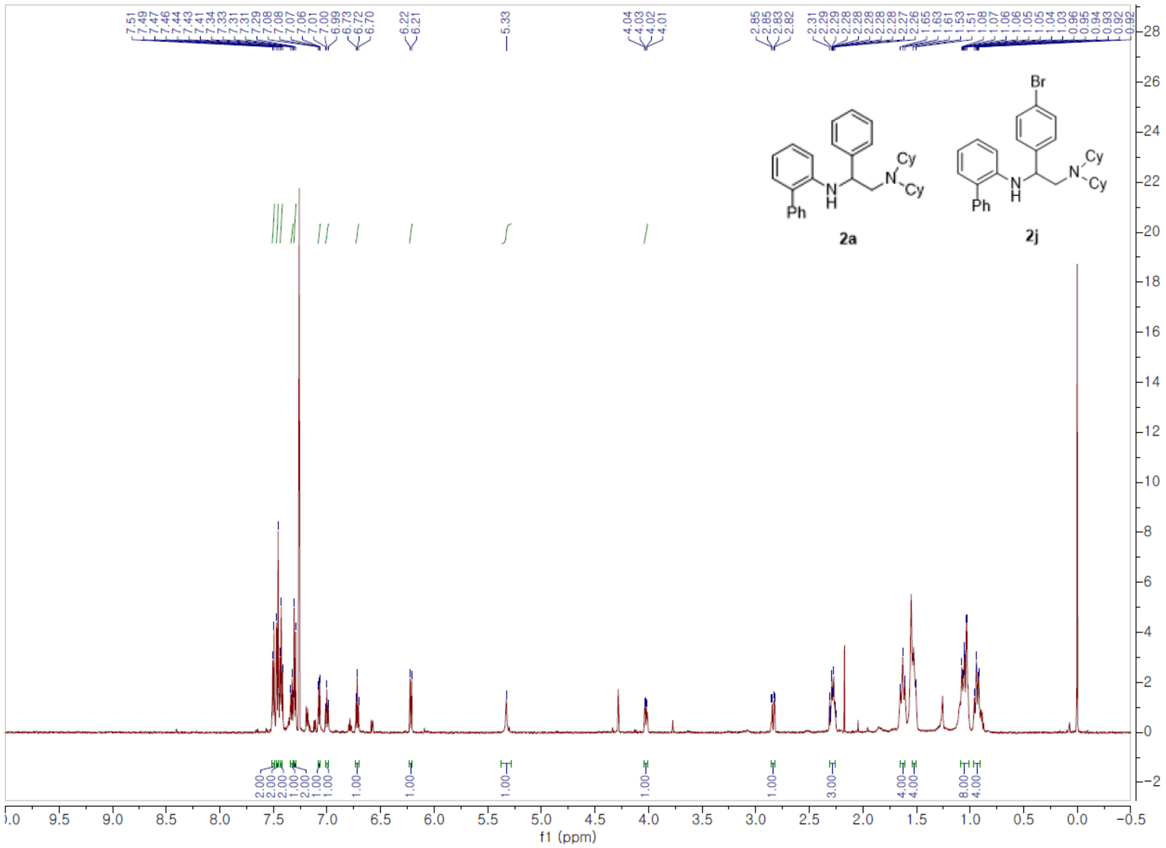

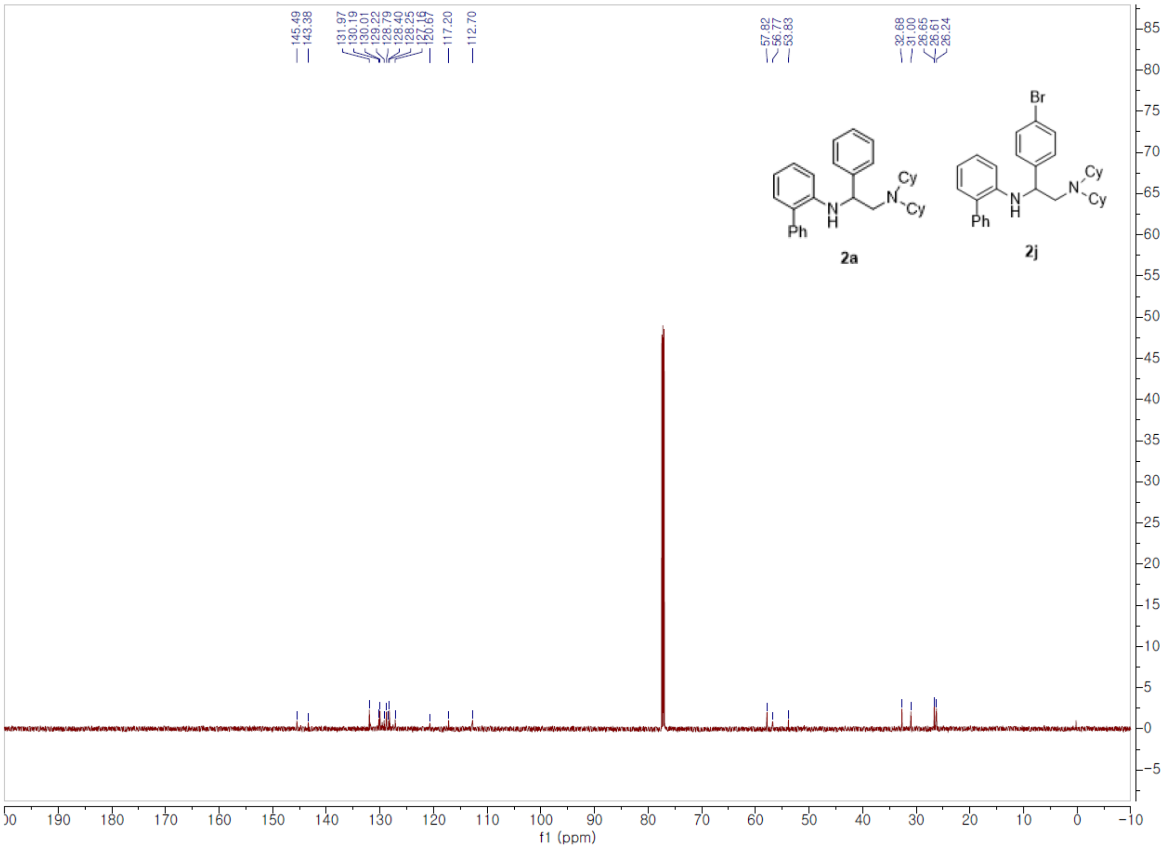

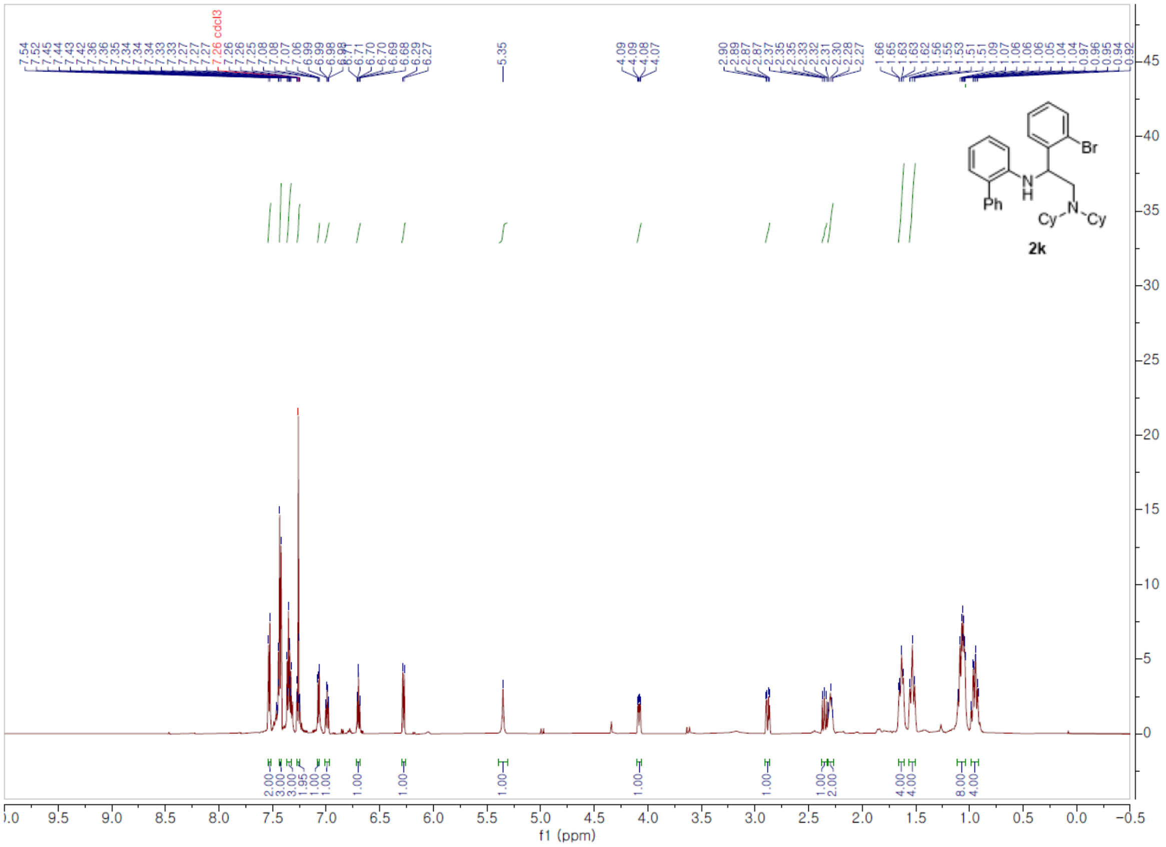

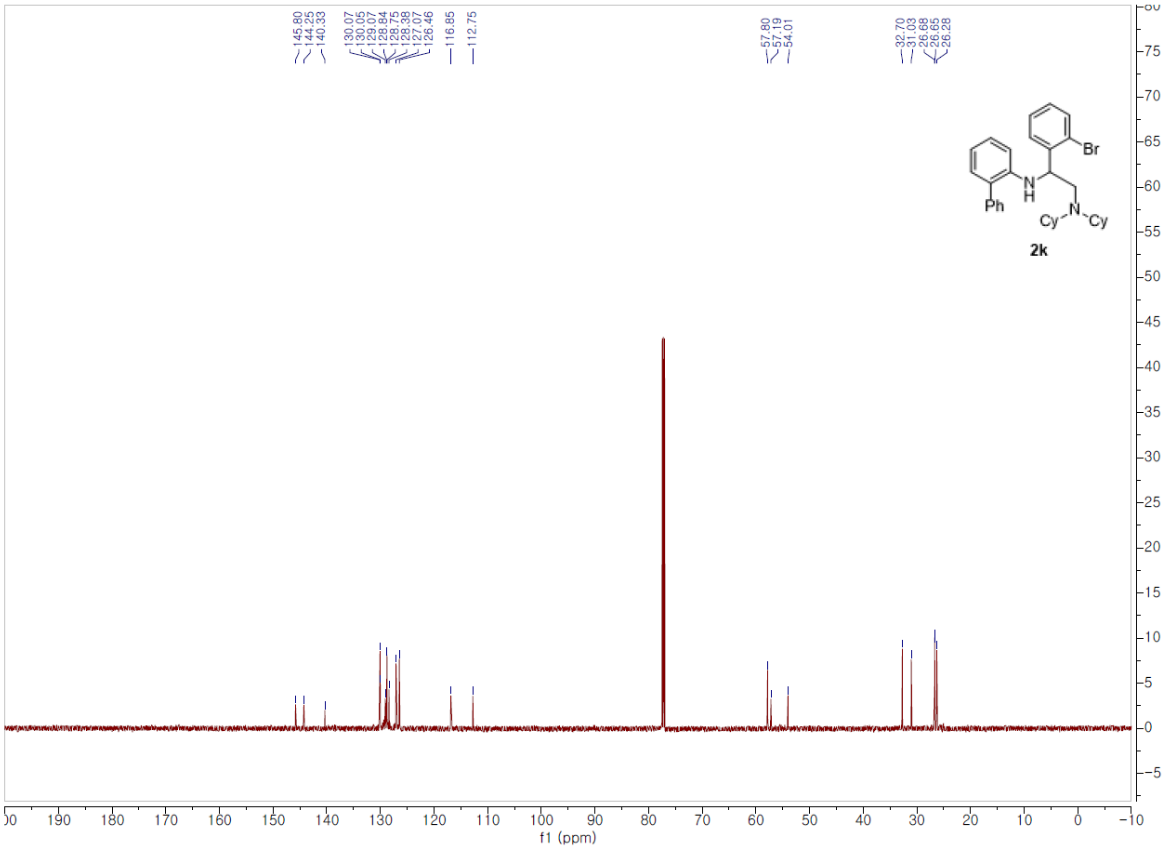

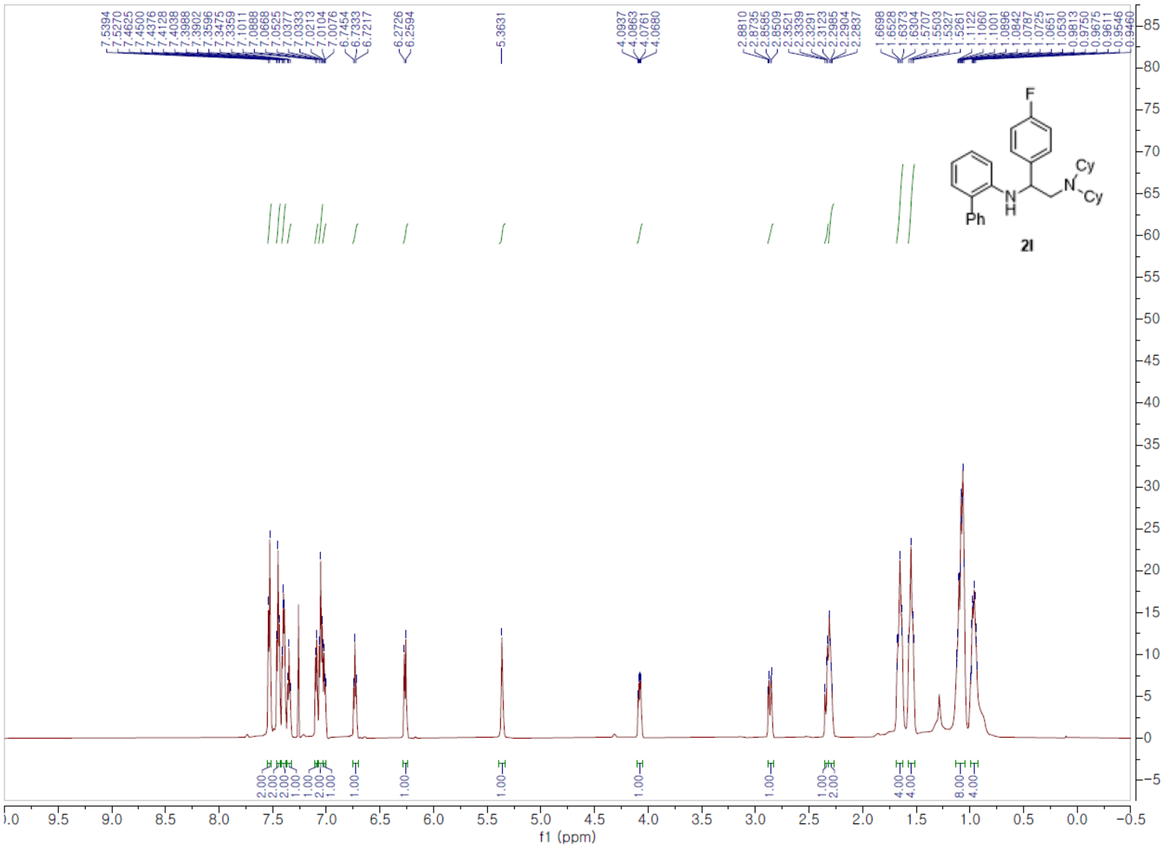
F
Cy
N
Cy
N
H
Ph
2l
f1 (ppm)

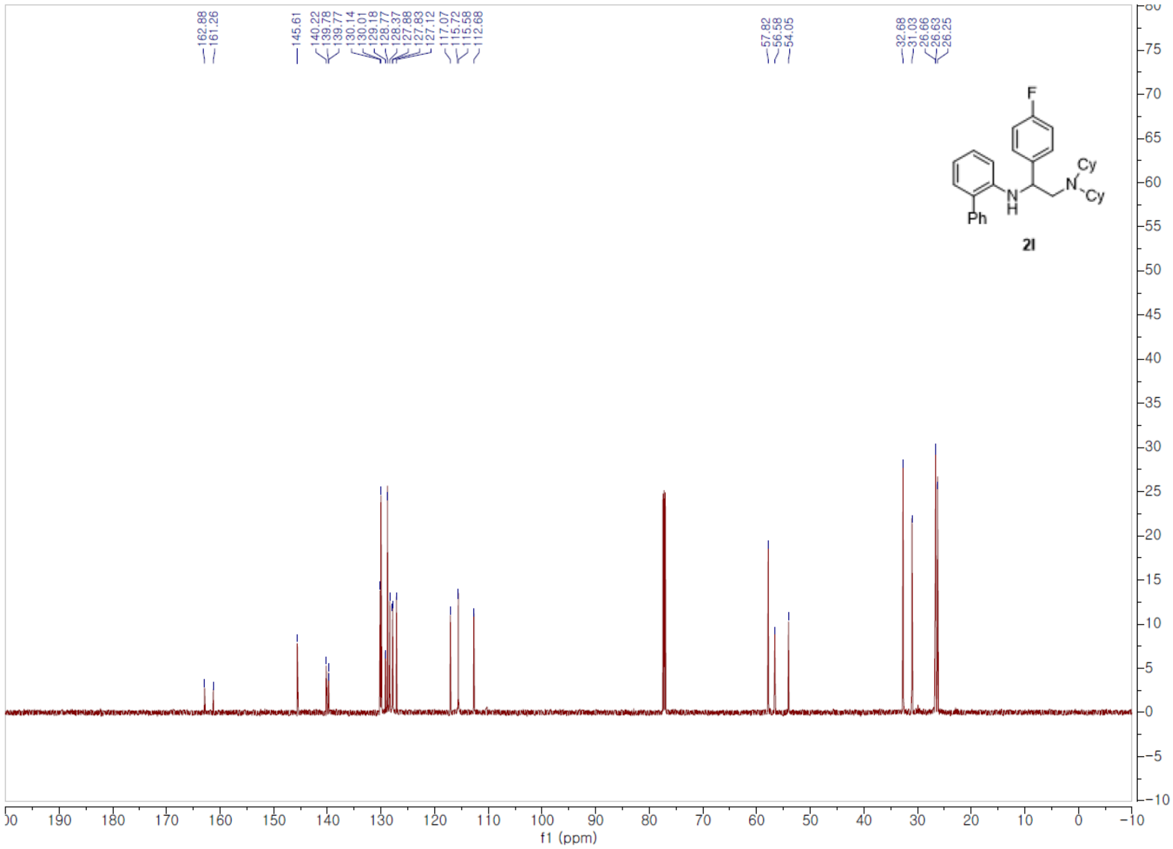
F
Cy
N
Cy
N
H
Ph
2l
f1 (ppm)

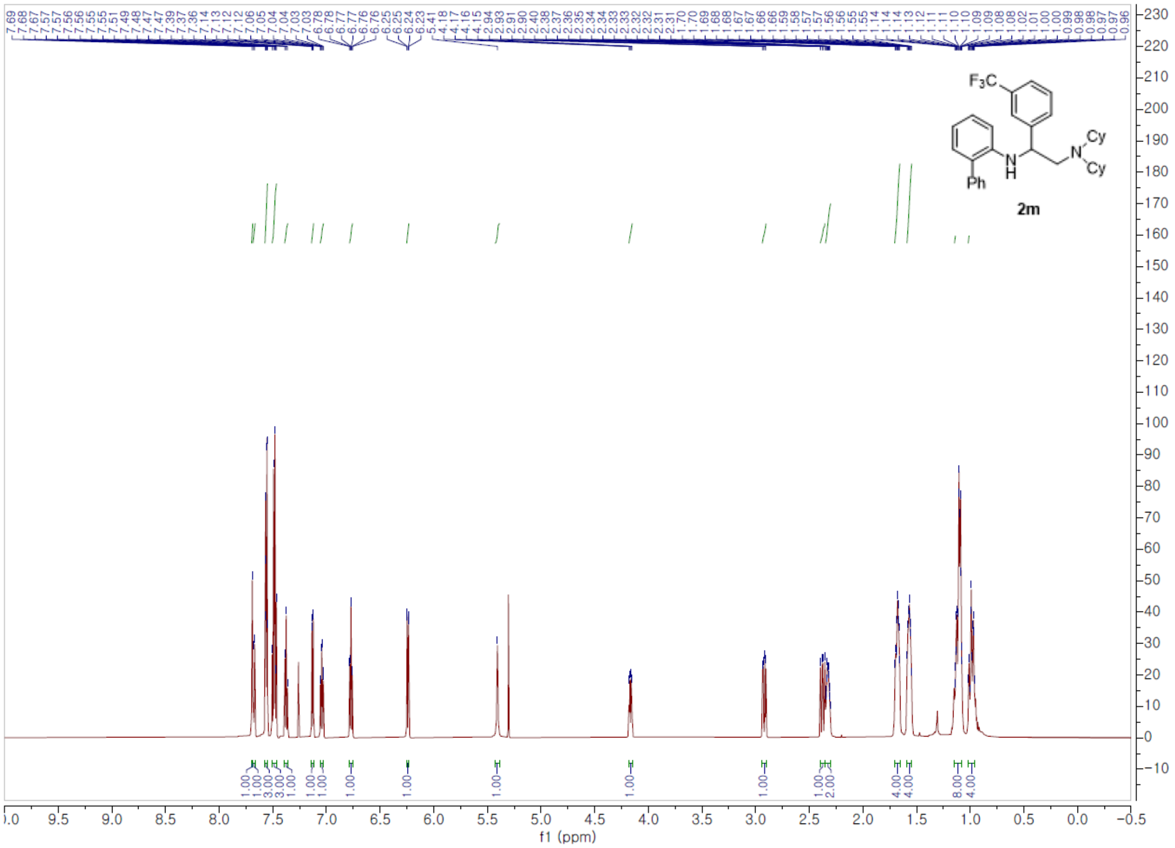
F3C
N
H
Ph
N
Cy
Cy
2m
f1 (ppm)

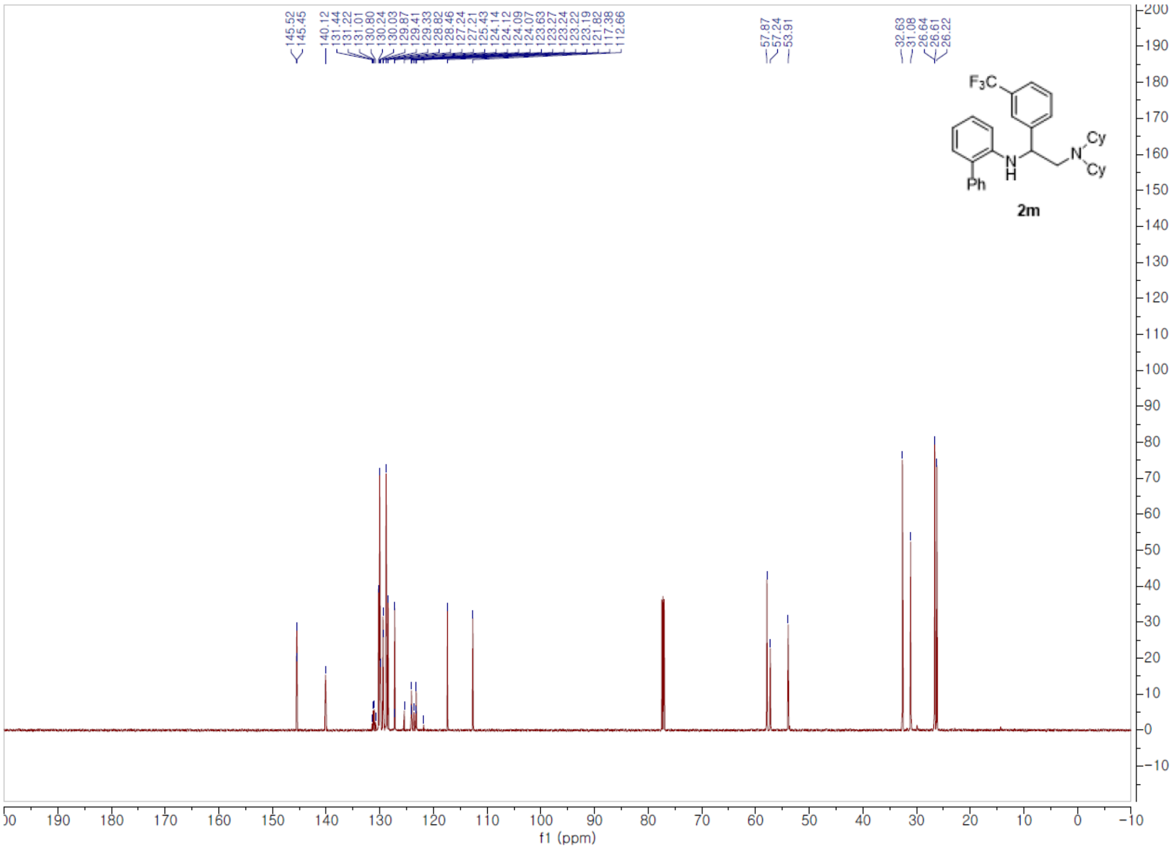
F3C
N
H
Ph
N
Cy
Cy
2m
f1 (ppm)

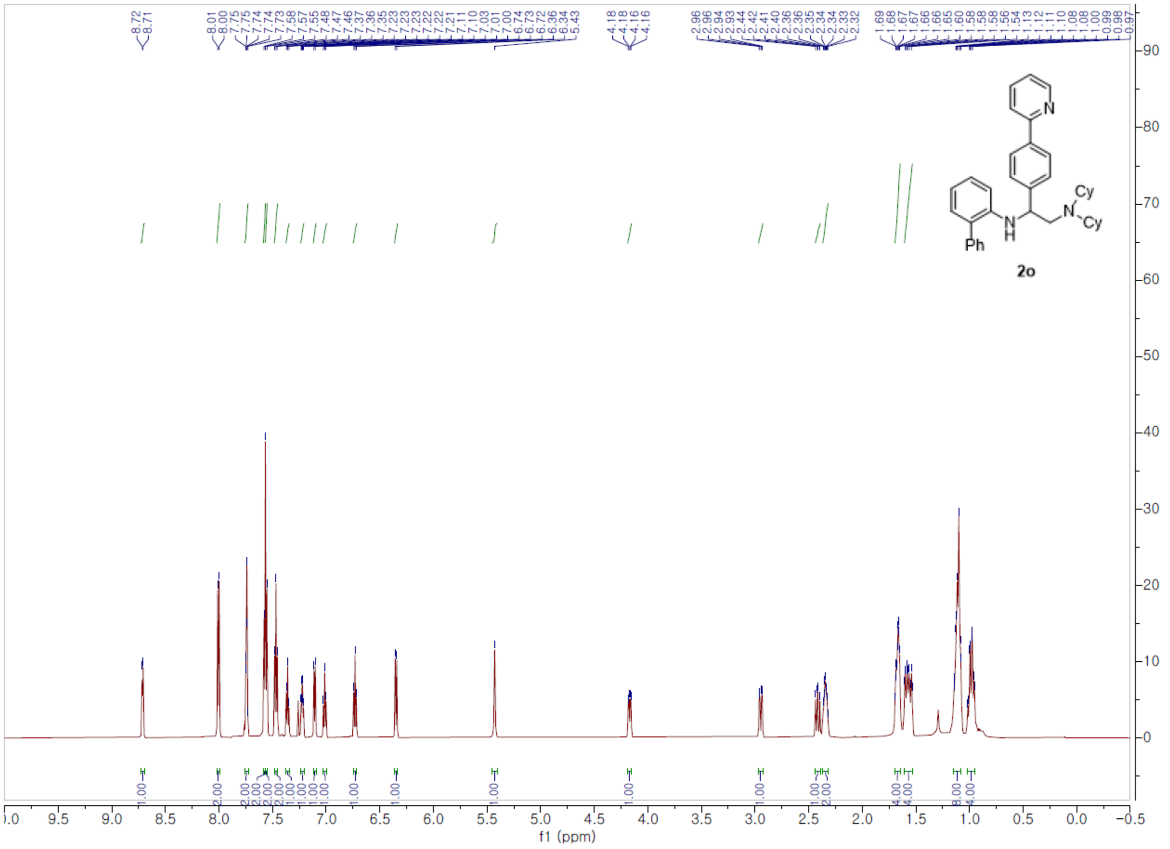
2o
f1 (ppm)

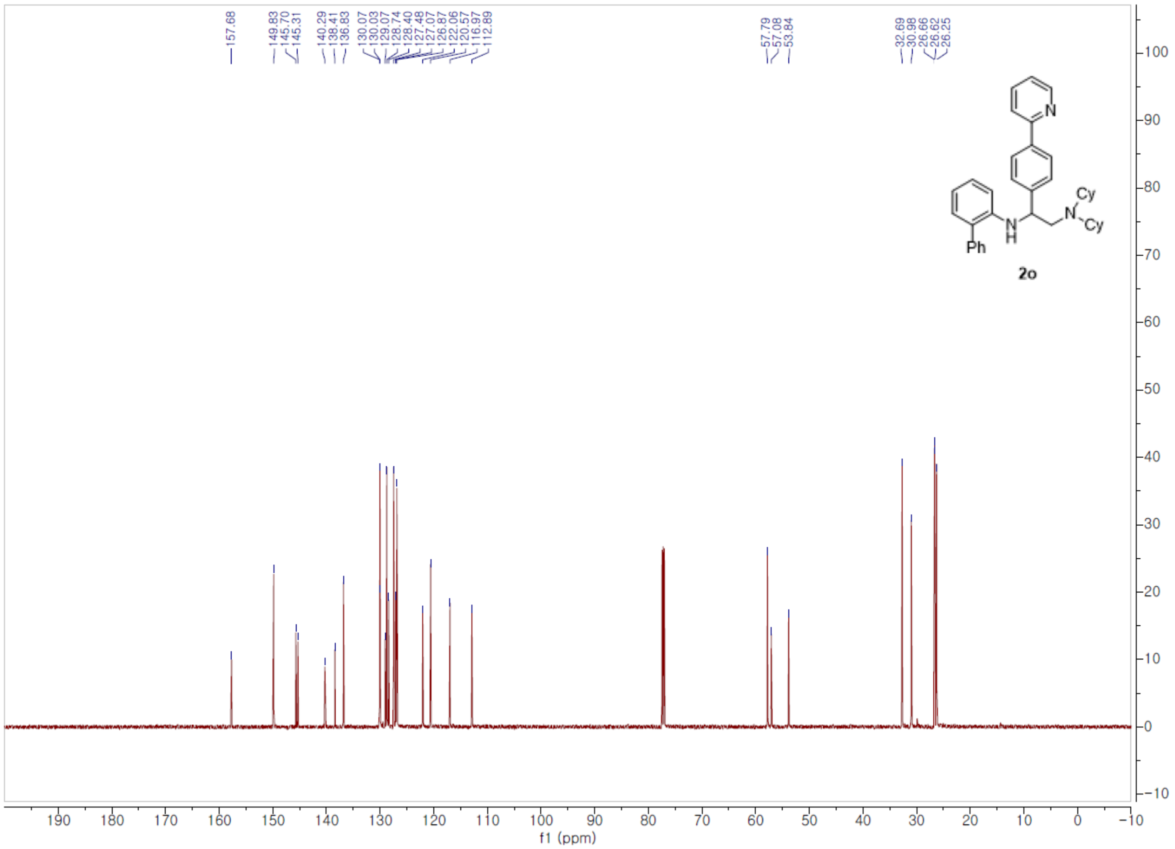
2o
f1 (ppm)

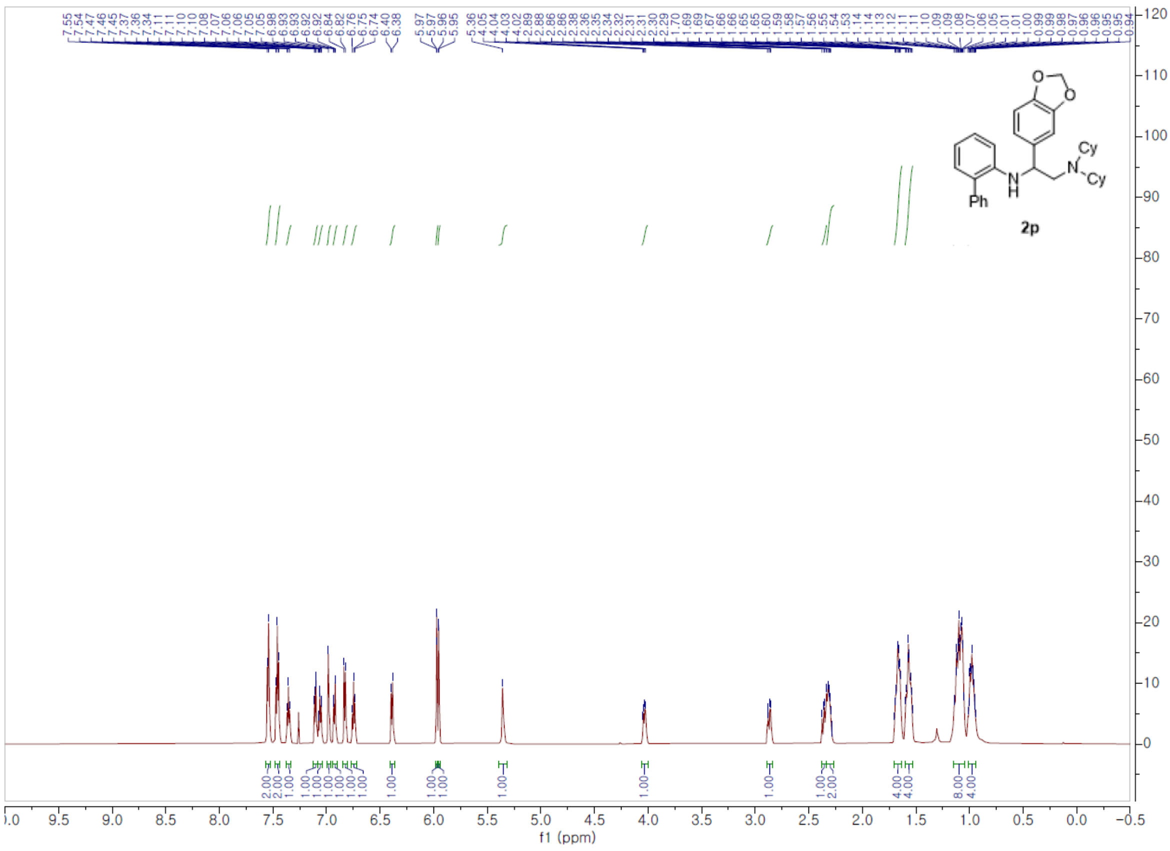

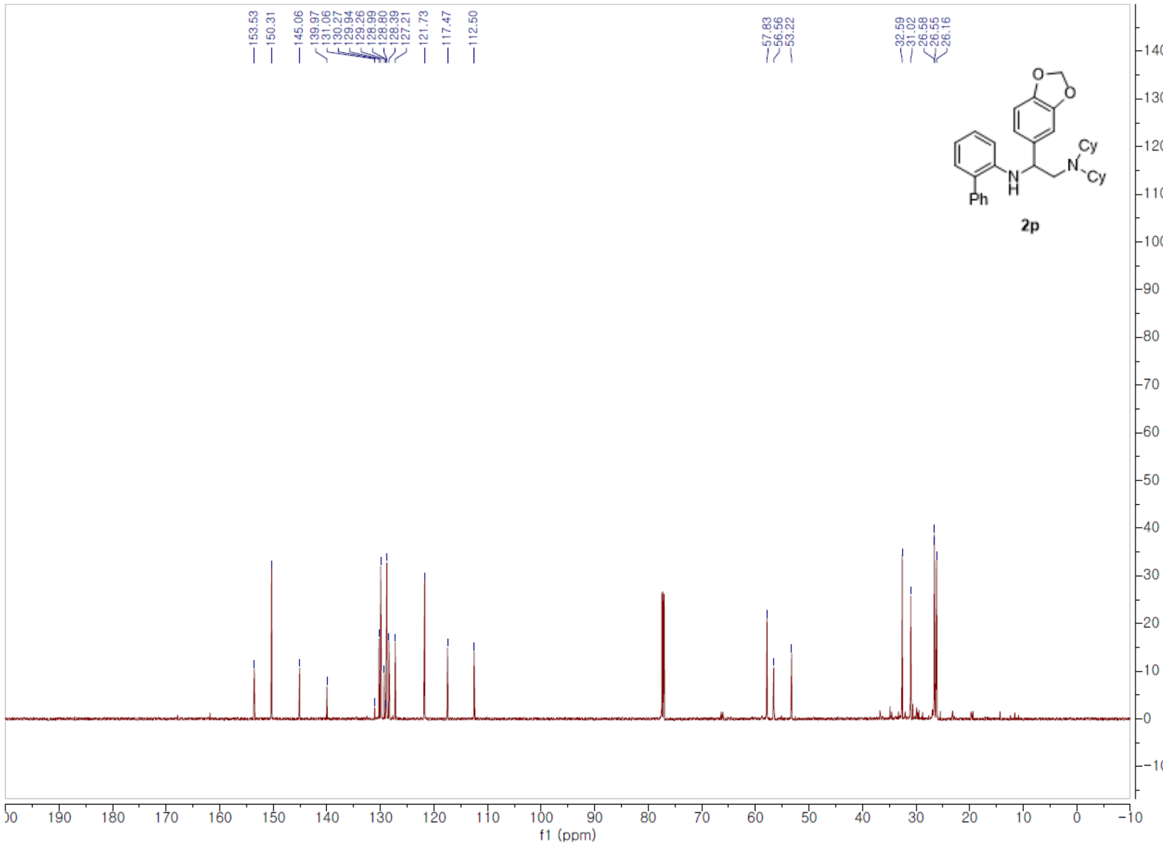

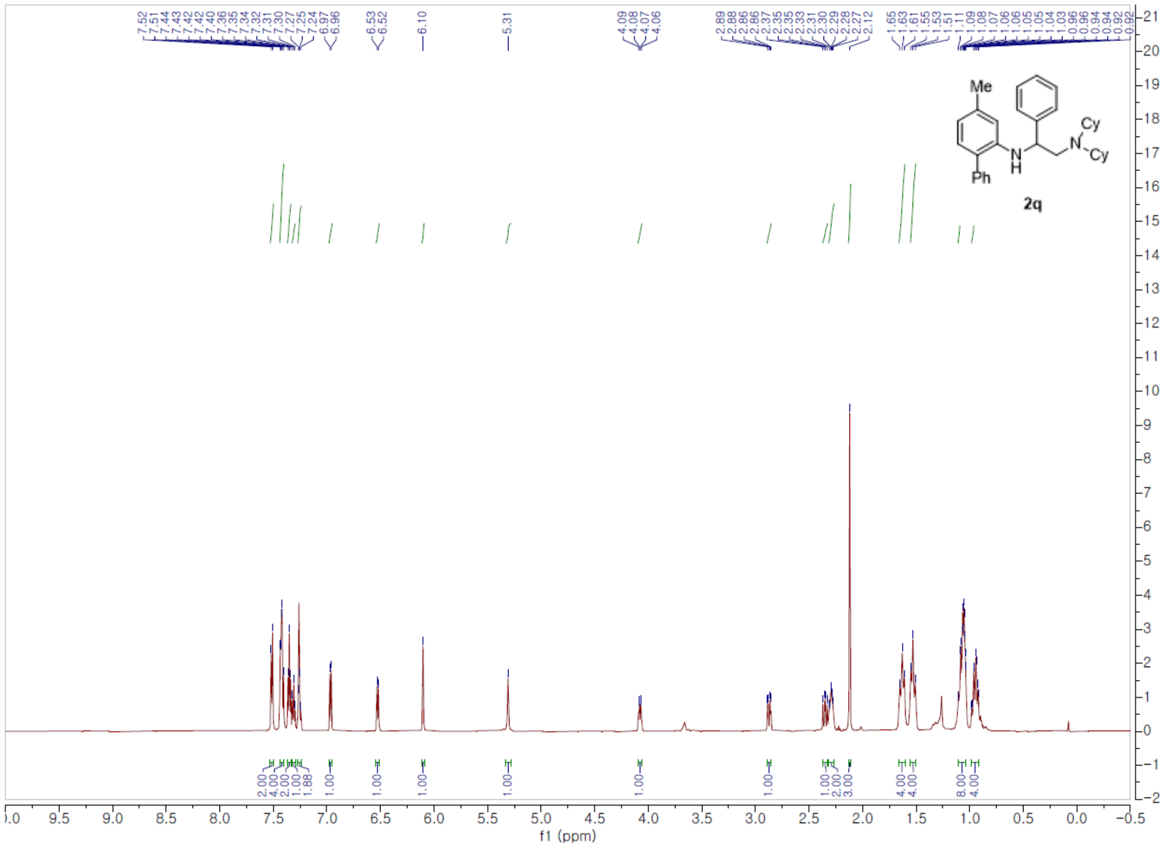

7.52
7.51
7.44
7.43
7.42
7.40
7.36
7.35
7.34
7.32
7.31
7.30
7.27
7.25
7.24
6.97
6.96
6.53
6.52
6.10
5.31
4.09
4.08
4.07
4.06
2.89
2.88
2.86
2.85
2.37
2.35
2.35
2.33
2.31
2.30
2.29
2.28
2.27
2.12
1.65
1.63
1.61
1.55
1.53
1.51
1.11
1.09
1.08
1.07
1.06
1.06
1.05
1.05
1.04
1.03
0.96
0.96
0.94
0.94
0.92
0.92
Me
N
H
Ph
Cy
N
Cy
2q
2.00
4.00
2.00
1.00
1.88
1.00
1.00
1.00
1.00
1.00
1.00
1.00
2.00
3.00
4.00
4.00
8.00
4.00
f1 (ppm)

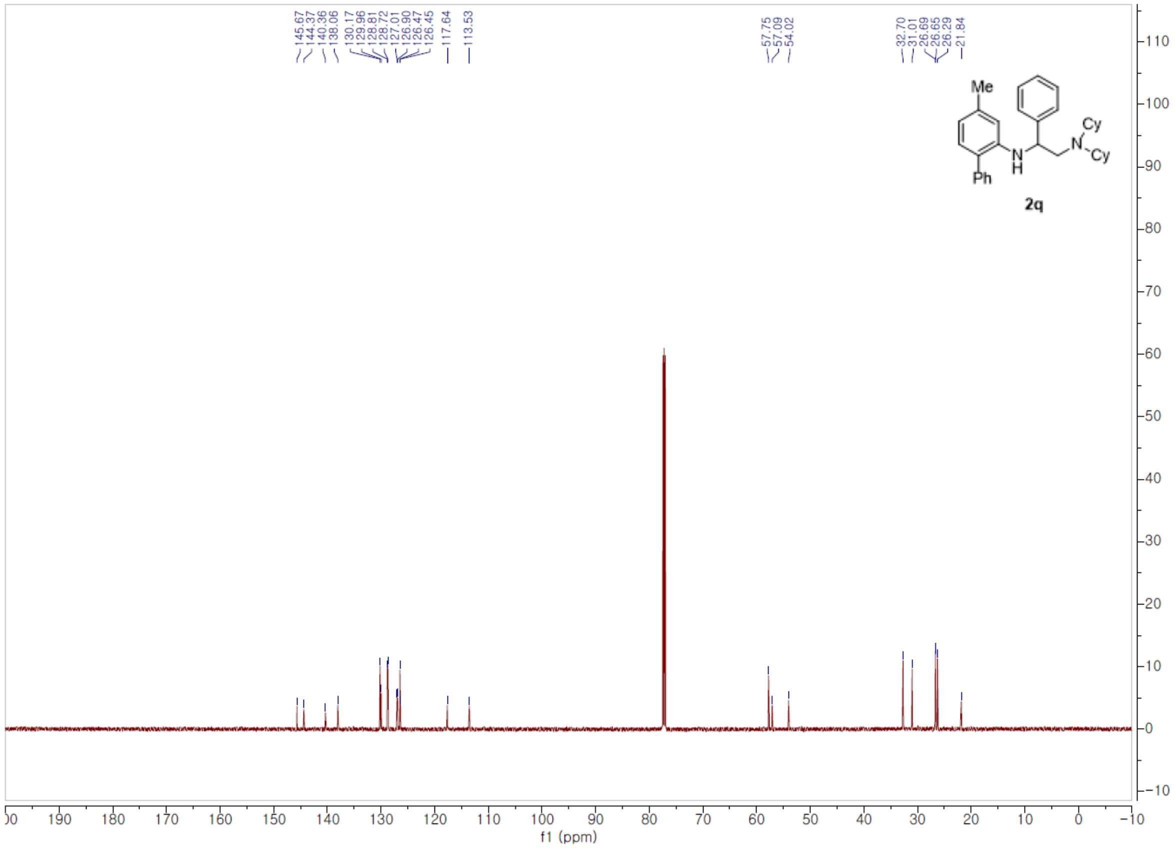

145.67
144.37
140.36
138.06
130.17
129.96
128.81
128.72
127.01
126.90
126.47
126.45
117.64
113.53
57.75
57.09
54.02
32.70
31.01
26.69
26.65
26.29
21.84
Me
N
H
Ph
Cy
N
Cy
2q
f1 (ppm)

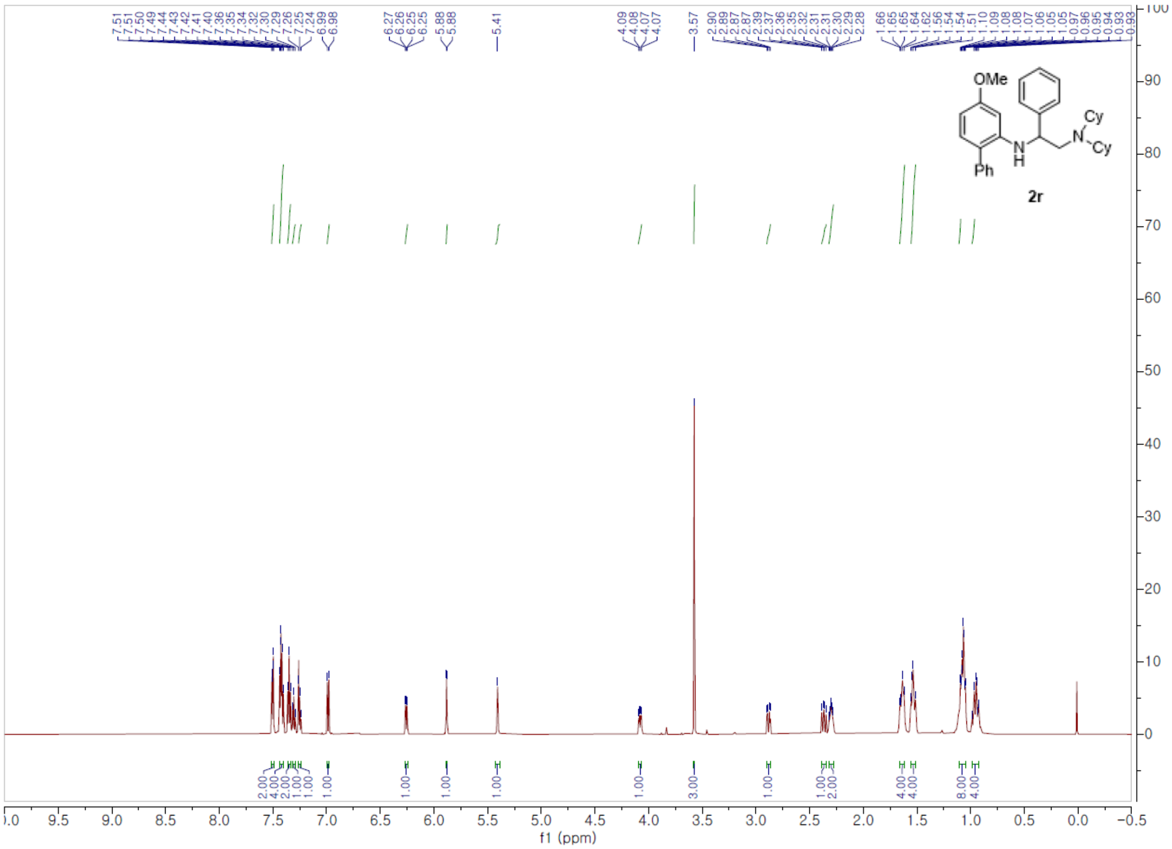
OMe
N H
Ph
Cy
N
Cy
2r
f1 (ppm)

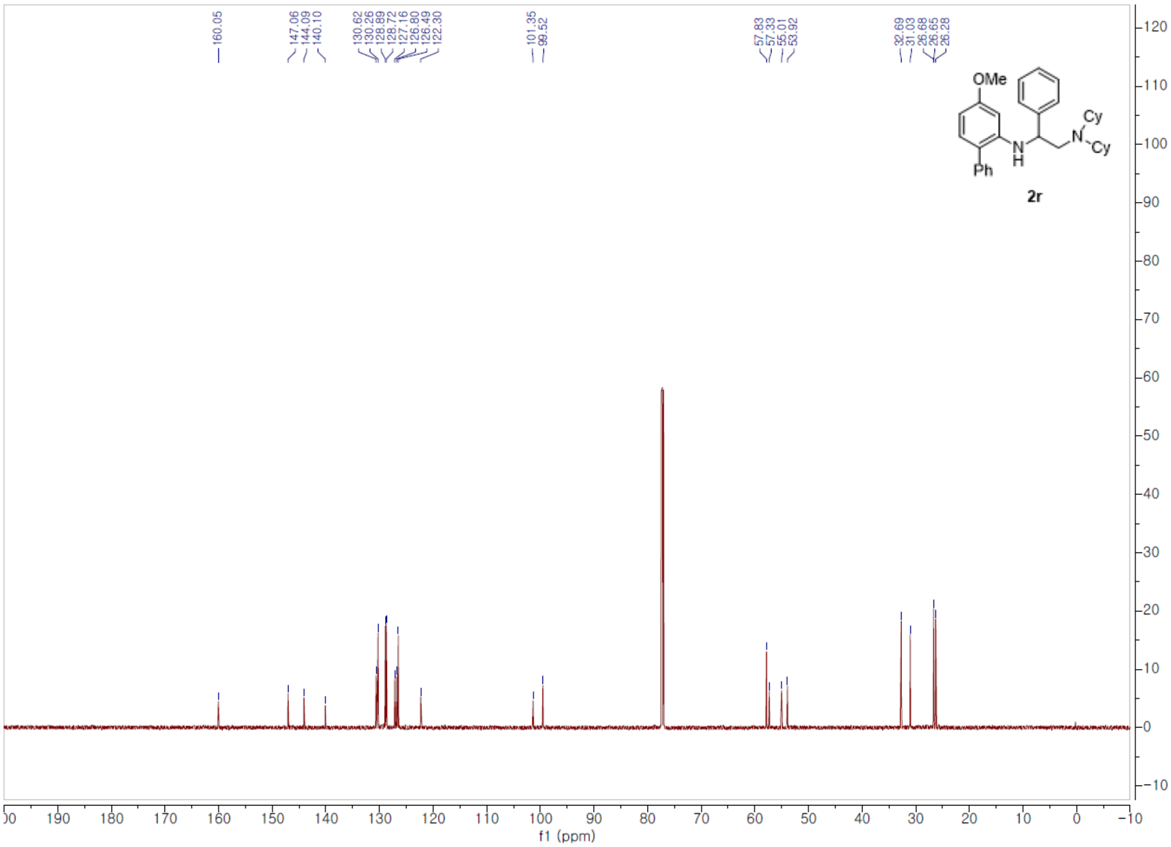
OMe
N H
Ph
Cy
N
Cy
2r
f1 (ppm)

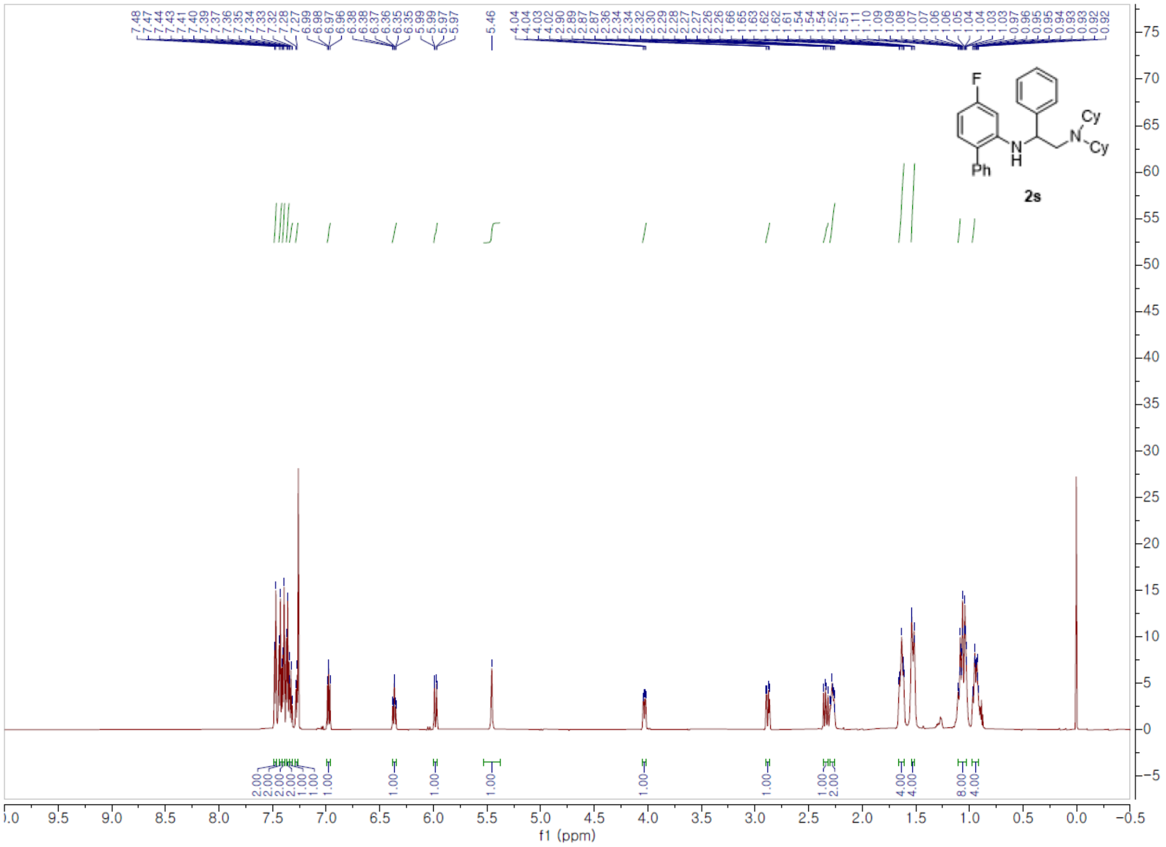

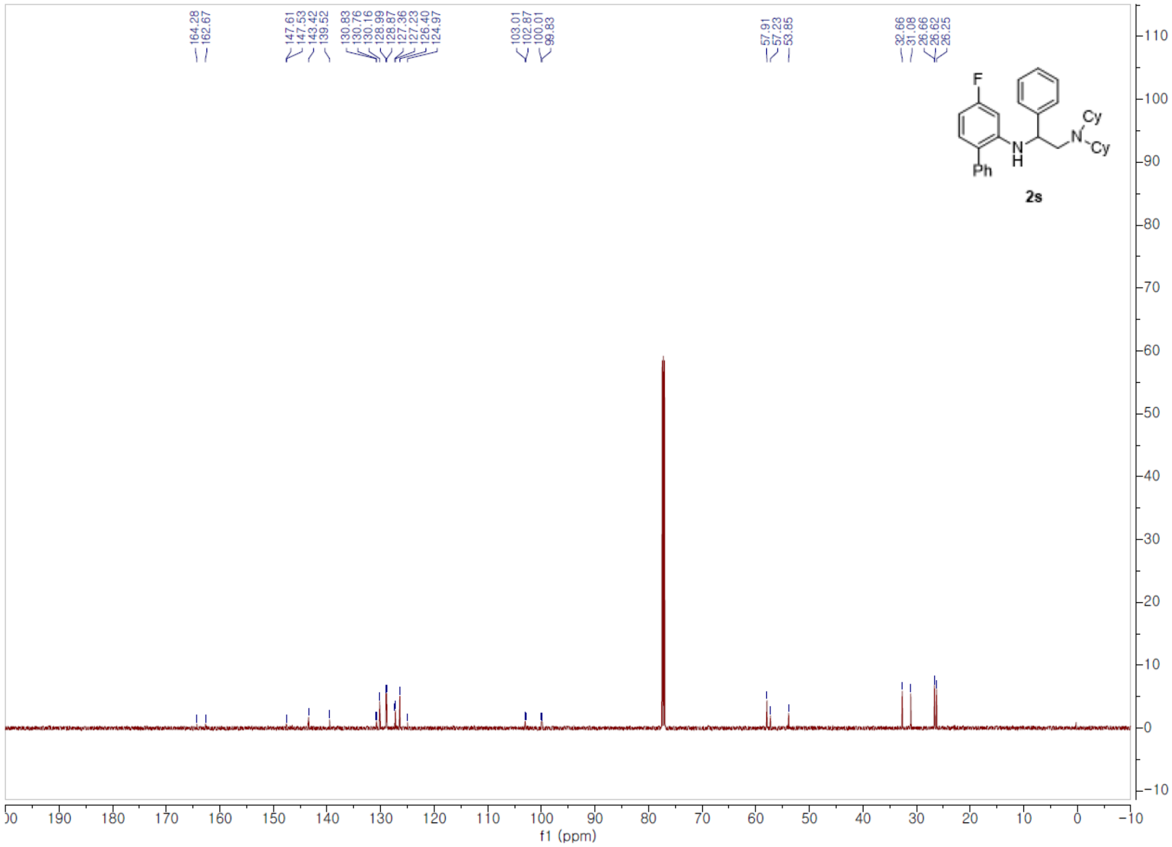

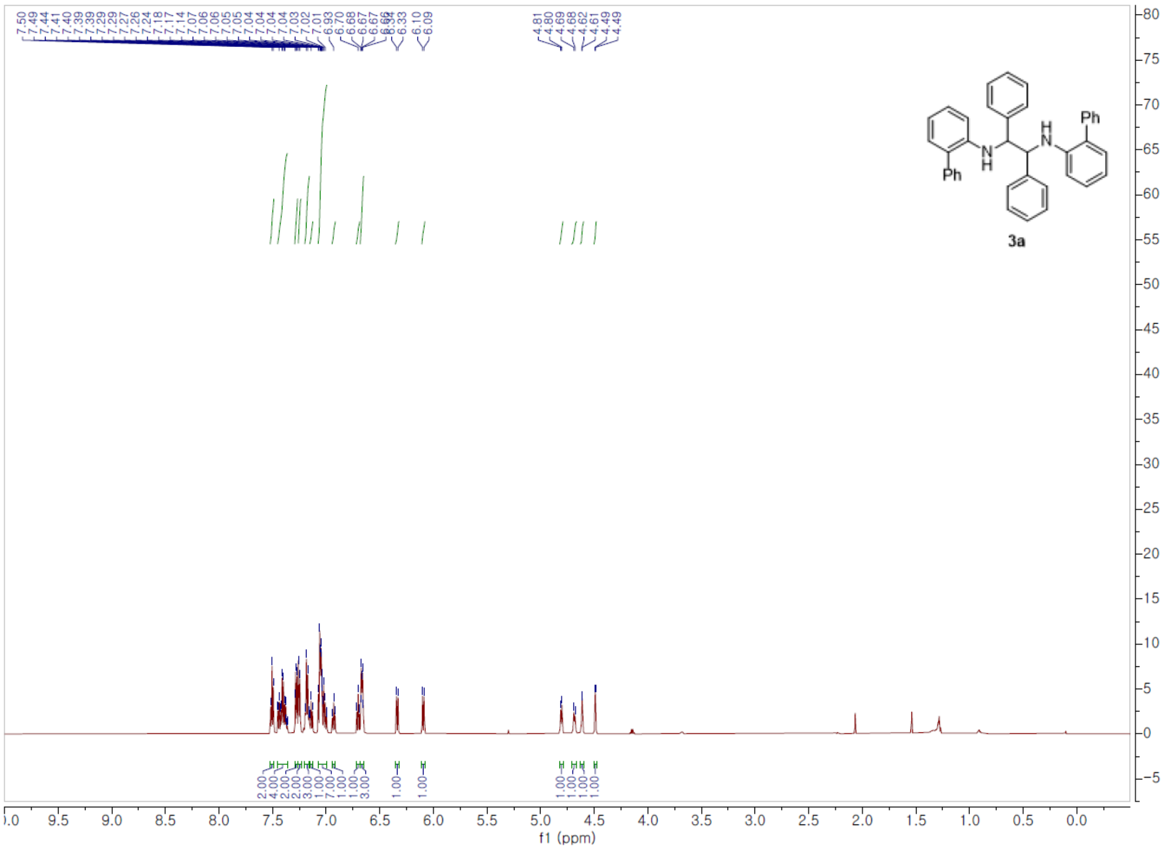

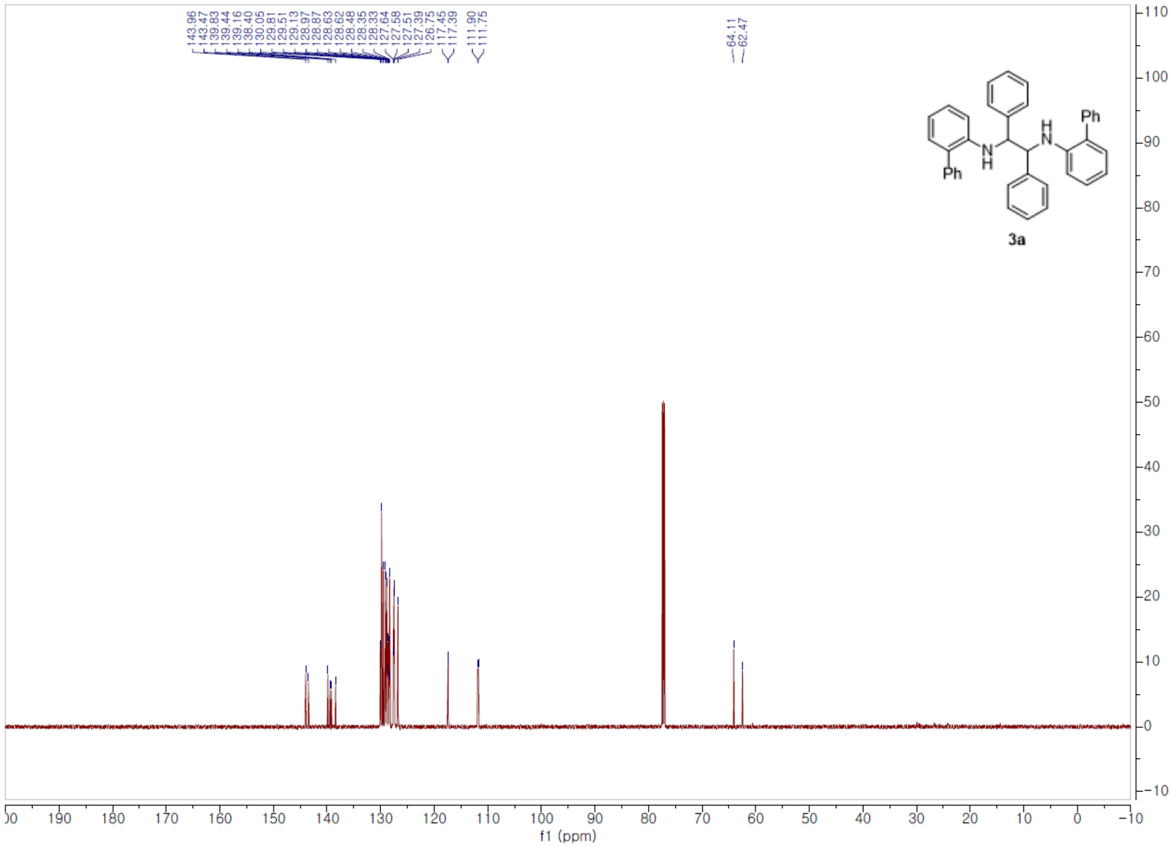

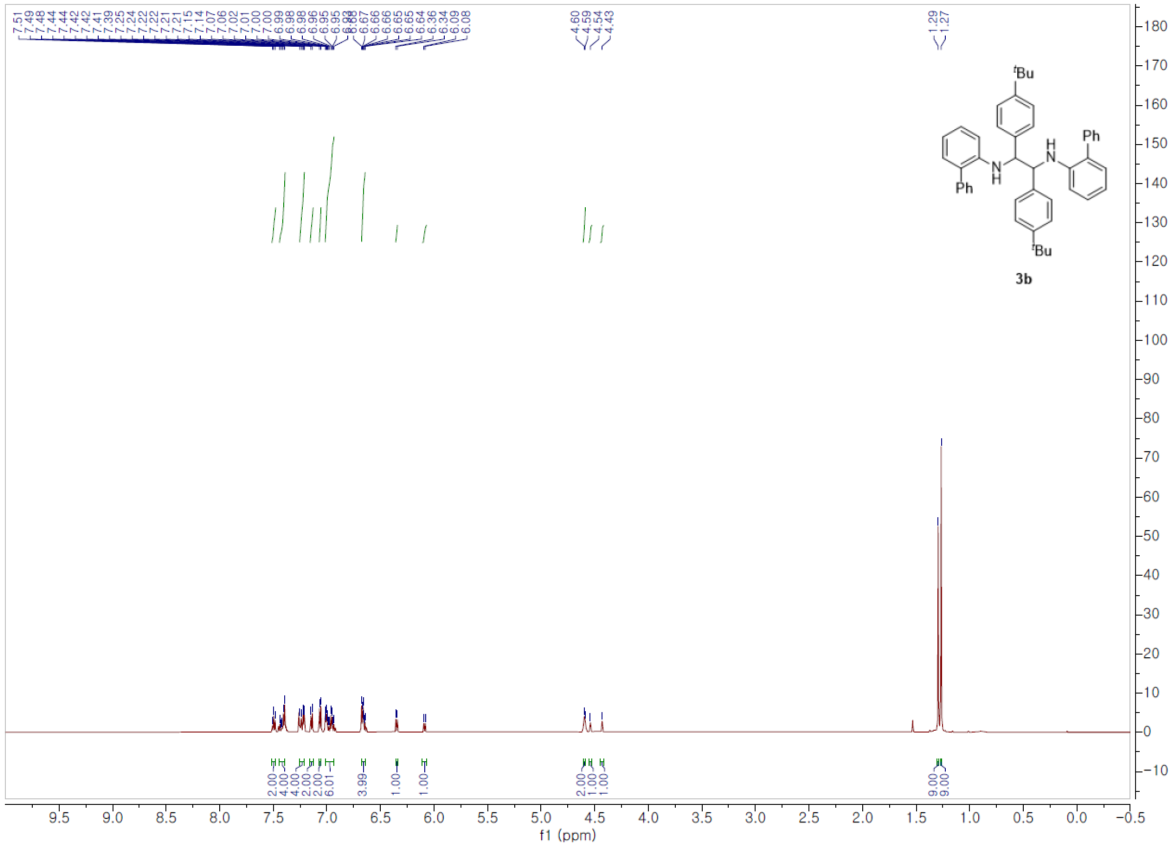
f1 (ppm)

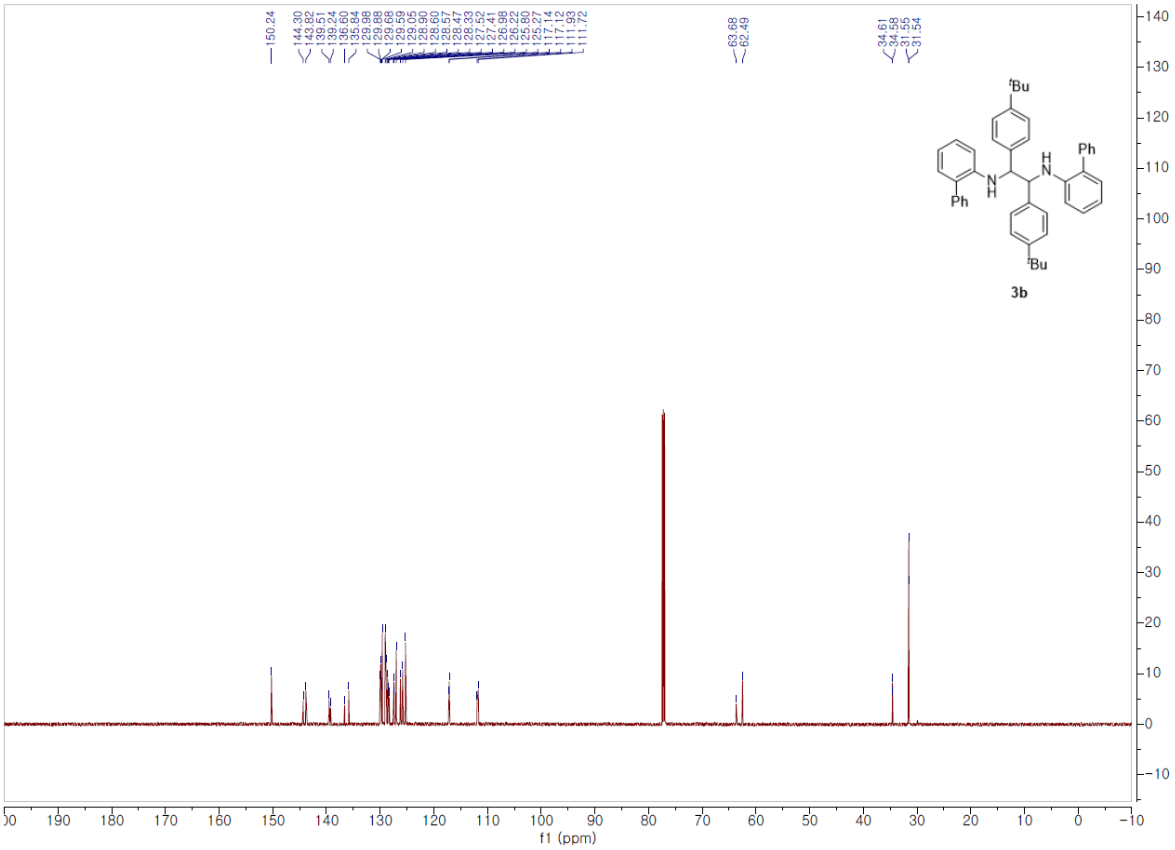
f1 (ppm)

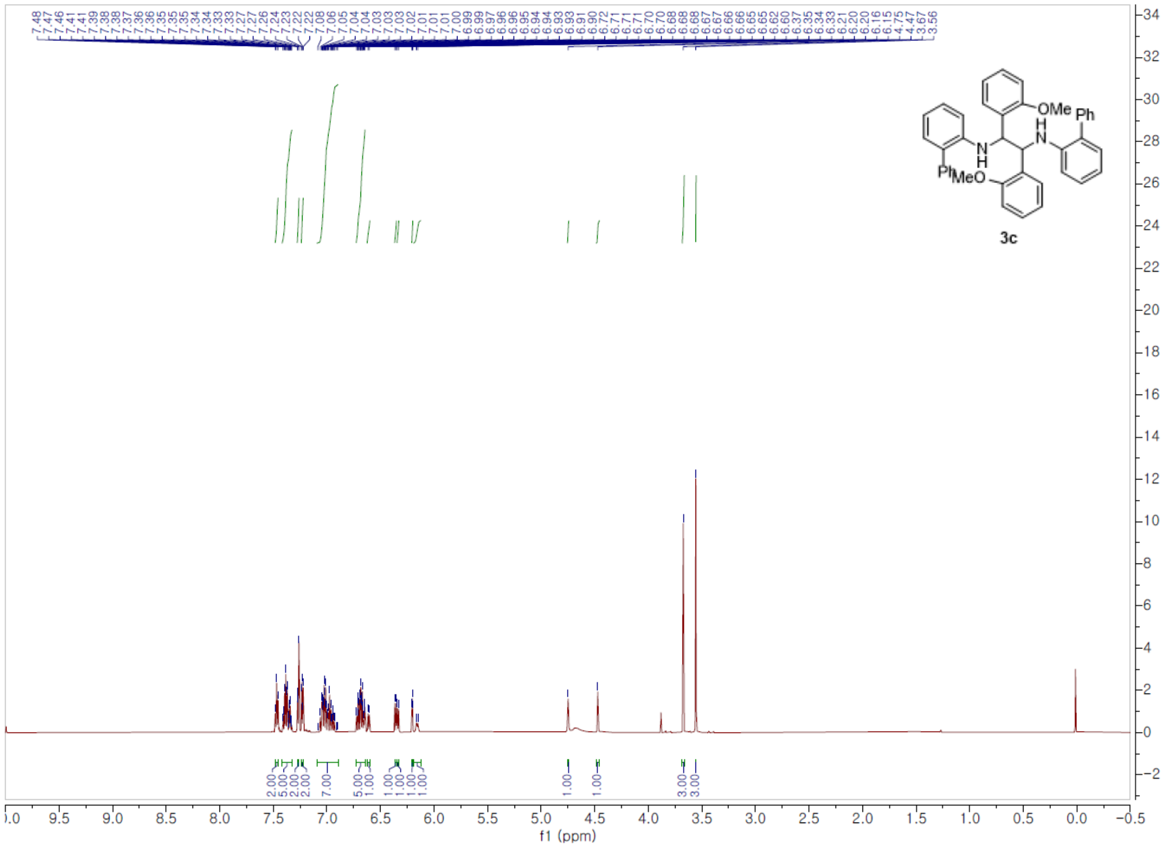

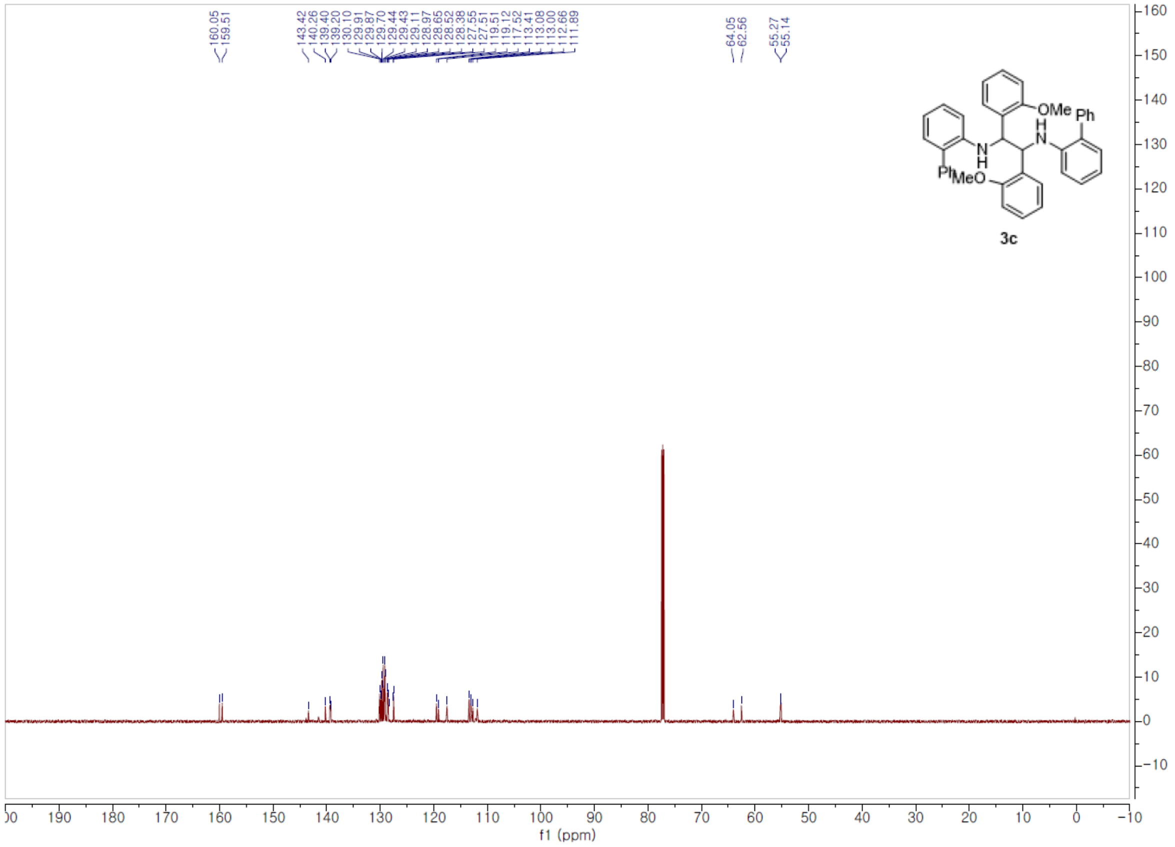

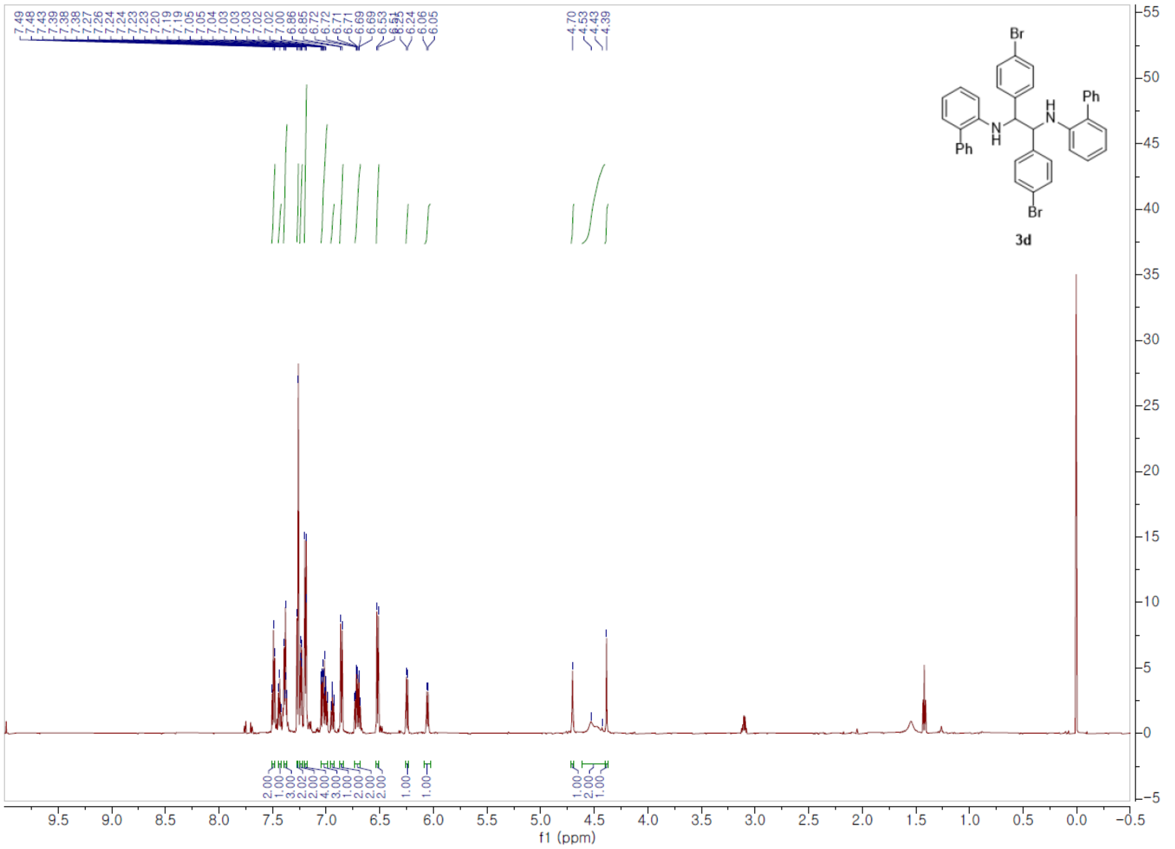

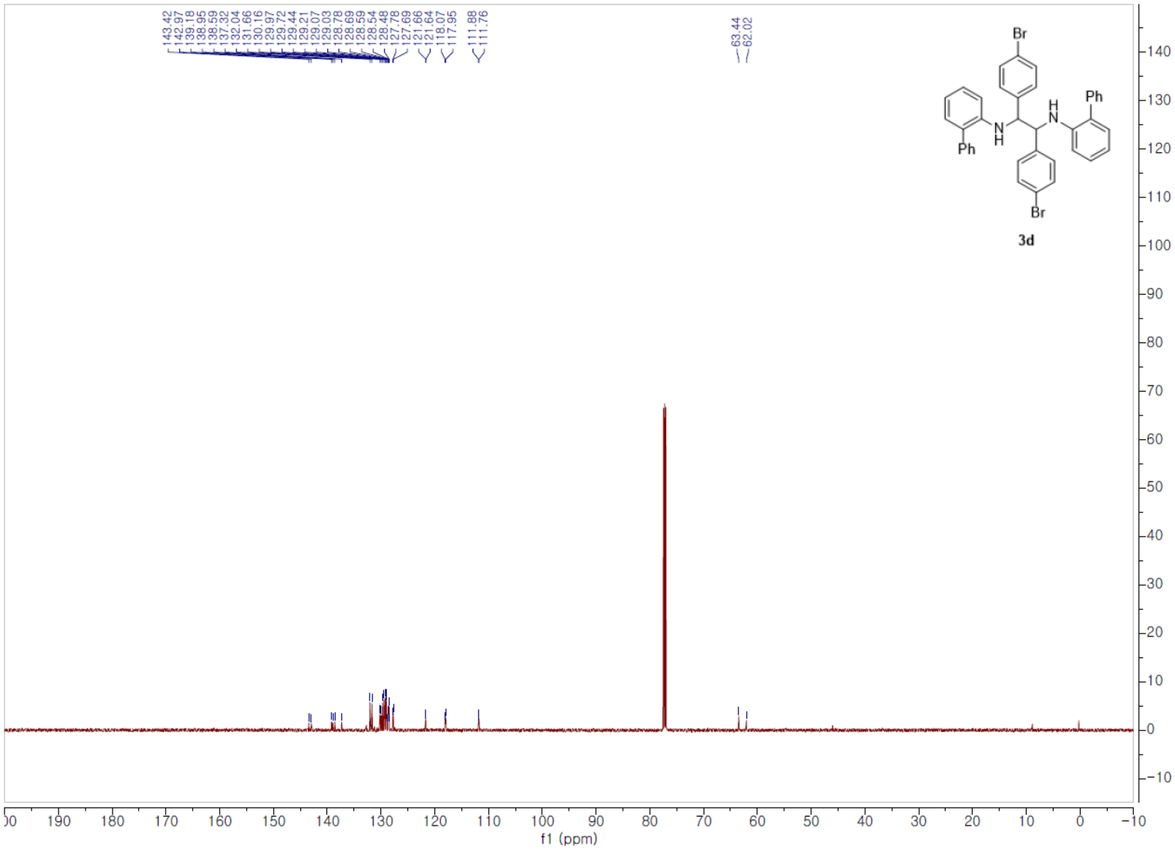

Supplement: File 1 — Additional experimental details and analytical data. [file Beilstein_J_Org_Chem-16-1335-s001.pdf]
